# Specialized dendritic cells induce tumor-promoting IL-10^+^IL-17^+^ FoxP3^*neg*^ regulatory CD4^+^ T cells in pancreatic carcinoma

**DOI:** 10.1038/s41467-019-09416-2

**Published:** 2019-03-29

**Authors:** Rocky M. Barilla, Brian Diskin, Raul Caso Caso, Ki Buom Lee, Navyatha Mohan, Chandan Buttar, Salma Adam, Zennur Sekendiz, Junjie Wang, Ruben D. Salas, Marcelo F. Cassini, Jason Karlen, Belen Sundberg, Hashem Akbar, Dmitry Levchenko, Inderdeep Gakhal, Johana Gutierrez, Wei Wang, Mautin Hundeyin, Alejandro Torres-Hernandez, Joshua Leinwand, Emma Kurz, Juan A. Kochen Rossi, Ankita Mishra, Miguel Liria, Gustavo Sanchez, Jyoti Panta, P’ng Loke, Berk Aykut, George Miller

**Affiliations:** 1 S. Arthur Localio Laboratory, Department of Surgery, New York University School of Medicine, 550 First Avenue, New York, NY 10016 USA; 2 Department of Microbiology, New York University School of Medicine, 550 First Avenue, New York, NY 10016 USA; 3 Department of Cell Biology, New York University School of Medicine, 550 First Avenue, New York, NY 10016 USA

## Abstract

The drivers and the specification of CD4^+^ T cell differentiation in the tumor microenvironment and their contributions to tumor immunity or tolerance are incompletely understood. Using models of pancreatic ductal adenocarcinoma (PDA), we show that a distinct subset of tumor-infiltrating dendritic cells (DC) promotes PDA growth by directing a unique T_H_-program. Specifically, CD11b^+^CD103^−^ DC predominate in PDA, express high IL-23 and TGF-β, and induce FoxP3^*neg*^ tumor-promoting IL-10^+^IL-17^+^IFNγ^+ ^regulatory CD4^+^ T cells. The balance between this distinctive T_H_ program and canonical FoxP3^+ ^T_REGS_ is unaffected by pattern recognition receptor ligation and is modulated by DC expression of retinoic acid. This T_H_-signature is mimicked in human PDA where it is associated with immune-tolerance and diminished patient survival. Our data suggest that CD11b^+^CD103^−^ DC promote CD4^+^ T cell tolerance in PDA which may underscore its resistance to immunotherapy.

## Abstract (web summary)

Pancreatic ductal adenocarcinoma is characterized by a highly immunosuppressive tumour microenvironment. Here, the authors show that specialized subsets of tumour-infiltrating dendritic cells induce distinct CD4^+^ T cell programs and specifically identify a CD103^–^CD11b^+^ subset which induces tumor-promoting FoxP3^–^ Type-1 regulatory T cells.

## Introduction

Autoimmunity denotes inflammatory responses directed against an organism’s own cells and tissues, and is driven by the systematic breakdown of the regulatory checkpoints governing cellular inhibition and self-tolerance. Peripheral tolerance can be mediated on a cellular level through the effector functions of distinct subsets of CD4^+^ T cells, including FoxP3^+^ T regulatory (T_REG_) cells and FoxP3^*neg*^ type-1 regulatory (Tr1) cells, or on a cell-intrinsic level through the upregulation of inhibitory receptors^1–3^. Since failure of these inhibitory processes can potentiate autoimmune responses against host antigens, it is not surprising that therapies targeting mechanisms of immune tolerance are being intensely investigated as potential treatments for cancer. Illustrating this is the recent advancement in checkpoint blockade and T-cell engineering, which has spurred a renaissance in cancer immunotherapy through approaches that override regulatory circuits to promote antitumor immunity^4^. Nonetheless, there are particular cancers, including pancreatic ductal adenocarcinoma (PDA), which respond very poorly to checkpoint blockade and adoptive T-cell therapy^5^. This may indicate the presence of a highly immunosuppressive tumor microenvironment (TME) that supports distinct, yet redundant, T-cell inhibitory programs. Alternatively, poor responses to immunotherapy may signify an obstruction in the stepwise process of T-cell priming by dendritic cells (DCs). Recent studies have described specialized subsets of TME-infiltrating antigen-presenting cells (APCs) distinguished by their unique abilities to prime, educate, and expand tumor-specific effector CD8^+^ T cells^6^. Antitumor cytotoxic T-cell responses are additionally influenced by fibrosis, infiltrating innate immune cells, and a number of TME-derived factors, all promoting immune tolerance through a variety of mechanisms^7–9^. Further, because of the complex repertoires of tolerogenic programs in select cancer subtypes, targeting CD8^+^ T cells alone may be insufficient to mount an adaptive immune response against specific tumors. As a result, ancillary methods of intervention may be required to consider T-cell-targeted therapy as a viable treatment modality for specific cancers.

Several autoimmune diseases (e.g., Crohn’s disease and psoriasis) have been linked to the imbalance of pathologic T_H_17 cells and tolerogenic T_REGS_^10–12^. In these diseases, the ultimate fate of CD4^+^ T-helper (T_H_) cell differentiation is attributed, at least in part, to the influence of DC from the site of inflammation^13^. While CD8^+^ T-cell priming by TME-infiltrating DC has been studied, we still have a limited understanding of (i) how tumor-infiltrating DCs direct CD4^+^ T_H_-cell differentiation and (ii) the functional roles differentiated T_H_ effector cells play in tumor progression. Furthermore, there is a lack of consensus on the role of TME-infiltrating T_H_17 cells in tumor progression, which may point to the functional complexity of this subset^14–16^. This discordance may stem from the de facto sufficiency of cytokine expression for classifying T-cell subsets without detailed functional analyses. The existence of both tolerogenic IL-17A^+^ T_REGS_ and immunogenic IL-17^+^ T_H_17 cells suggests that IL-17^+^ T_H_ cells may represent several functionally distinct subsets^17^. As cytotoxic CD8^+^ effector function is highly dependent on CD4^+^ T-cell cooperation, exploration of cellular and biochemical drivers T_H_-cell differentiation may hold promise for making resistant cancers more immunogenic. As such, we investigated the effect of DC education on T_H_-cell programming and immune tolerance in the PDA TME.

## Results

### PDA-infiltrating DC direct CD4^+^ T-cell differentiation and promote disease progression

**Fig. 1:**
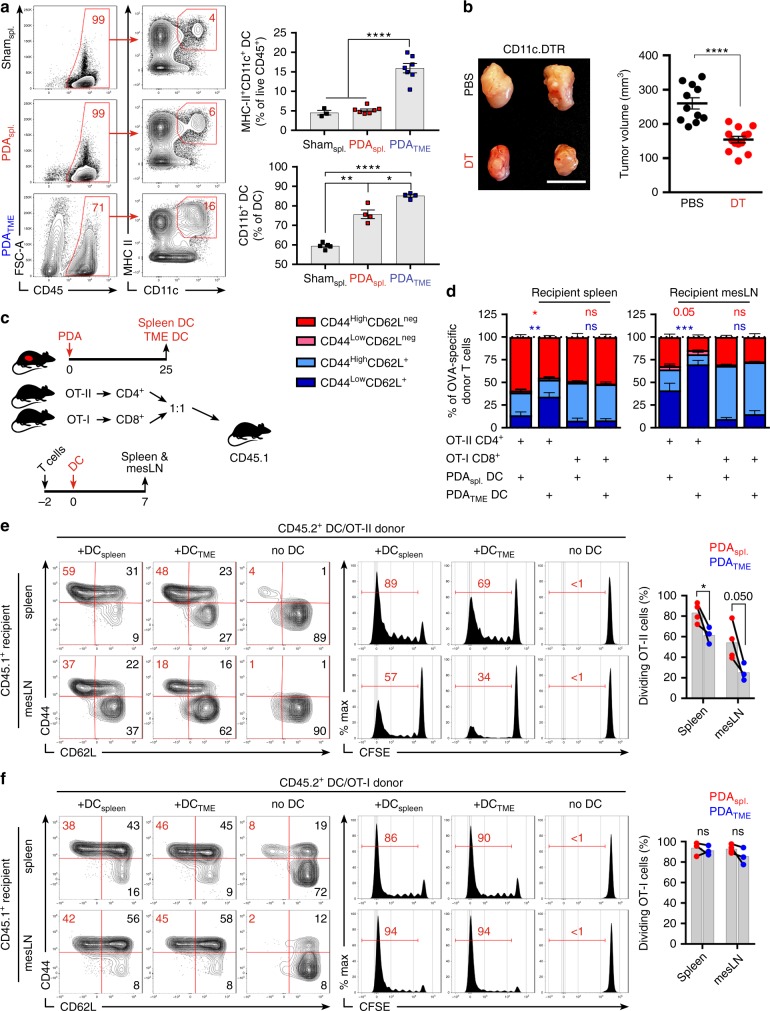
Distinct pancreatic ductal adenocarcinoma (PDA)-infiltrating dendritic cell (DC) promote tumor progression and regulate CD4^+^ T-cell differentiation. **a** CD45^+^ cells from the spleen or tumor of orthotopic PDA-bearing mice or from the spleen of control mice were gated using flow cytometry and tested for co-expression of CD11c and MHCII. CD11c^+^MHCII^+^ cells were then sub-gated and tested for expression of CD11b. Representative contour plots and quantitative data are shown. This experiment was repeated >5 times. **b** WT mice were made chimeric using bone marrow from CD11c.DTR mice. Cohorts were challenged with orthotopic PDA 7 weeks later. On day 12, mice began serial treatment with diphtheria toxin (DT) or phosphate-buffered saline (PBS) before sacrifice on day 25. Representative gross images of tumors and quantitative analysis of tumor volume are shown. This experiment had similar results for >5 times (scale bars = 1 µm). **c**–**f** PDA_TME_ DC were harvested on day 25 from tumor-bearing mice, loaded with either Ova_323–339_ or Ova_257–264_ peptide and administered in equal number i.p. to CD45.1 mice that had been transferred i.v. 2 days prior with equal numbers of CFSE-labeled OT-I and OT-II T cells. Parallel experiments were performed using PDA_spl._ DC. On day 7 after either PDA_TME_ or PDA_spl_ DC.Ova administration, spleen and mesenteric lymph nodes (LNs) were harvested and CD45.2^+^ CD4^+^ and CD8^+^ T cells tested for co-expression of CD44 and CD62L and dilution of CFSE. **c** A schematic of the experimental regimen is depicted. **d** Quantitative results and **e** representative flow cytometry data for CD4^+^ T cells and **f** CD8^+^ T cells are shown. This experiment was repeated twice (*p < 0.05, **p < 0.01, ***p < 0.001, ****p < 0.0001, *t*-test), SEM

Along with others, we have shown that CD4^+^ T cells are ineffective at generating antitumor immunity in PDA^18–20^. We postulated that select DC subsets within the TME entrain CD4^+^ T cells towards a tolerogenic phenotype. Approximately 15% of CD45^+^ leukocytes infiltrating primary PDA tumors in mice were CD11c^+^MHCII^+^ (PDA_TME_ DC) (Fig. 1a). The percentage of DC in the spleens of PDA-bearing mice (PDA_spl._) was similar to control spleen (sham_spl._); however, PDA_spl._ DC contained a greater CD11b^+^ fraction (Fig. 1a). To investigate the influence of DC on tumor progression, we utilized CD11c.DTR bone marrow chimeric mice, which allowed for serial depletion of DC after PDA establishment (Supplementary Figure 1A-B). Macrophage and T-cell infiltration and macrophage phenotype were unchanged after DC depletion (Supplementary Figure 1C, D). DC depletion resulted in marked tumor protection (Fig. 1b). DC depletion was similarly protective in a PDA liver metastases model (Fig. S1E). We hypothesized that PDA_TME_ DC facilitate tumor progression by inducing tolerogenic CD4^+^ or CD8^+^ T-cell differentiation. To test this, we performed an in vivo CD4^+^ and CD8^+^ T-cell expansion assay by adoptively transferring carboxyfluorescein *N*-succinimidyl ester (CFSE)-labeled antigen-restricted CD4^+^ and CD8^+^ T cells from OT-II and OT-I donors, respectively, into congenic CD45.1 mice followed by the transfer of Ova-pulsed DC from either the spleen or TME (Fig. 1c). Splenic and nodal CD45.2^+^CD4^+^ T cells in hosts receiving antigen-pulsed PDA_TME_ DC exhibited diminished activation—evidenced by a smaller CD44^high^CD62L^neg^ population—and a lower proliferative response than in mice primed by Ova-pulsed splenic DC (Figs. 1d, e). In addition, while interferon-γ (IFNγ) was similarly expressed by antigen-restricted CD4^+^ T cells in mice transferred with spleen or TME-derived DC, the expression of interleukin (IL)-17A was significantly greater in CD4^+^ T cells primed by TME-derived DC (Supplementary Figure 1F). These observations were not a result of DC immaturity or inability to capture antigen as PDA_TME_ DC expressed significantly higher CD86, ICOS-ligand (ICOSL), CCR7, and MHCII and captured antigen more avidly than splenic DC subsets (Supplementary Figure 1G and H). The elevated expression of IL-17A in antigen-restricted CD4^+^ T cells primed by PDA_TME_ DC paralleled the phenotype of CD4^+^ T cells in situ in PDA, which expressed greater IL-17A than splenic CD4^+^ T cells in PDA-bearing mice (Supplementary Figure 1I). To determine whether CD4^+^ T-cell expression of IL-17 in situ in PDA was DC dependent, we depleted DC in PDA-bearing CD11c.DTR bone marrow chimeric mice. Ablation of DC mitigated CD4^+^ T-cell expression of IL-17 and retinoic acid receptor-related orphan receptor gamma t (RORγt) (Supplementary Figure 1J). Ablation of DC similarly reduced CD4^+^ T-cell expression of IL-17 and RORγt in the PDA liver metastases model (Supplementary Figure 1K). In contrast to the differential effects of PDA_TME_ DC on CD4^+^ T-cell differentiation, the proliferation and activation of antigen-restricted CD8^+^ T cells did not differ between hosts primed with PDA_TME_ DC versus splenic DC (Figs. 1d, f), suggesting distinct influences of PDA_TME_ DC on CD4^+^ T-cell differentiation. To definitively determine whether the tumor-protective effects of DC depletion were CD4^+^ T-cell dependent, we ablated DC in PDA-bearing hosts in the context of CD4^+^ T-cell depletion. Absence of CD4^+^ T cells negated the tumor-protective effect of DC depletion, suggesting that the protection afforded by DC ablation was CD4^+^ T-cell dependent (Supplementary Figure 2A). By contrast, DC depletion offered residual protective effects in absence of CD8^+^ T cells (data not shown). This led us to believe that PDA_TME_ DC may constrain CD4^+^ T-cell-driven antitumor responses and motivated us to further investigate their interactions.

### PDA_TME_ DC induce a distinct CD4^+^ T-cell program

**Fig. 2:**
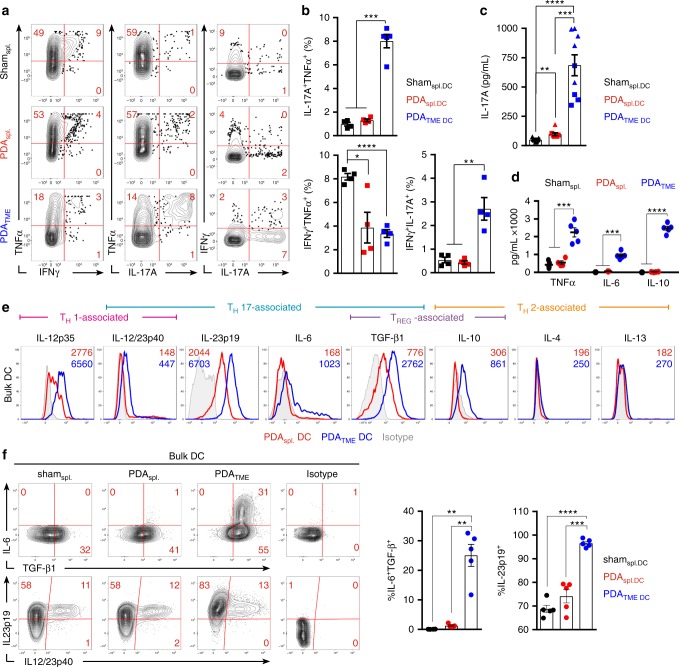
PDA_TME_ dendritic cell (DC) exhibit a unique cytokine profile and promote IL-17 expression in naive CD4^+^ T cells. **a**–**c** Ova_323–339_ peptide-pulsed PDA_TME_ DC and splenic DC controls were co-cultured with Ova-restricted CD4^+^ T cells at a 1:5 ratio for 5 days. Representative (**a**) and quantitative (**b**) intracellular cytokine expression in the CD4^+^ T cells are shown. Data are representative of five independent experiments using 3–6 mice per arm, showing similar results. **c** Concentration of IL-17A in the supernatants of co-cultures were determined. Squares and triangles denote data points from individual mice, pooled from two independent experiments. **d** PDA_TME_ DC and splenic DC controls were plated at a density of 5 × 10^5^ cells/ml without re-stimulation and supernatant cytokine concentrations were determined after 36 h in a cytometric bead array. Data are representative of experiments repeated >3 times. **e** Orthotopic pancreatic ductal adenocarcinoma (PDA) tumors and spleens were harvested from mice on day 25 post-implantation. Intracellular flow cytometric analysis of DC cytokine expression after 12 h of re-stimulation with LPS (1 μg/ml) is shown. Representative histograms are shown. Numbers represent median fluorescence indices (MFI). Data are representative of experiments repeated >3 times. **f** The frequency of IL-6, TGF-β1 and IL23p19, IL12/23p40 co-expression in PDA_TME_ DC and splenic DC controls were tested. Representative contour plots and quantitative data are shown (*n* = 5 per group). This experiment was repeated three times (*p < 0.05, **p < 0.01, ***p < 0.001, ****p < 0.0001, *t*-test), SEM

To determine how PDA entrainment affects DC capacity for T_H_-cell differentiation, we co-cultured Ova-pulsed PDA_TME_ DC and controls with Ova-restricted CD4^+^ T cells. Consistent with our in vivo data, PDA_TME_ DC possessed enhanced capacity for the induction of IL-17 as evidenced by increased generation of IL-17A^+^TNFα^+^ and IL-17A^+^IFNγ^+^ CD4^+^ T cells (Figs. 2a, b; Supplementary Figure 2B). By contrast, splenic DC groups were not efficient at inducing IL-17 expression in CD4^+^ T cells. The T_H_ phenotype induced by PDA_TME_ DC was verified through the assessment of secreted cytokines, which exhibited the highest concentrations of T_H_17-associated cytokines IL-17A and IL-6, and lowest concentrations of IL-2 compared with splenic DC-CD4^+ ^T-cell co-cultures (Fig. 2c; Supplementary Figure 2C). CD4^+^ T-cell expression of IL-10 was also differentially induced by PDA_TME_ DC (Supplementary Figure 2C). Consistent with the cytokine profile of PDA_TME_ DC-entrained CD4^+^ T cells, Ova-pulsed PDA_TME_ DC induced more RORγt^+^ and FoxP3^+^ Ova-restricted CD4^+^ T cells but fewer T-bet^+^ CD4^+^ cells than did control DC populations (Supplementary Figure 2D and E). We confirmed these results in an alternate model of T-cell activation as PDA_TME_ DC promoted the secretion of greater IL-17A and IL-6, but less IL-2, in mixed lymphocyte reactions (MLRs) with allogeneic CD4^+^ T cells (Supplementary Figure 2F). Similarly, IL-17-rich cytokine profiles were observed in antigen-restricted CD4^+^ T cells co-cultured with antigen-pulsed PDA_TME_ DC that were harvested from both autochthonous *P48*^*Cre*^*;Kras*^*G12D*^ (KC) and *Pdx1*^*Cre*^*;Kras*^*G12D*^*;Tp53*^*R172H*^ (KPC) mouse PDA tumors, suggesting these observations are not specific to the orthotopic PDA model (Supplementary Figure 2G, H).

Because CD4^+^ T-cell differentiation can be driven by secreted inflammatory mediators, we examined the cytokines produced by PDA_TME_ DC. Purified PDA_TME_ DC produced markedly elevated TNFα, IL-6, and IL-10 in culture compared with splenic DC from tumor-bearing hosts (Fig. 2d). Further, intracellular cytokine analysis suggested that PDA_TME_ DC expressed elevated levels of additional T_H_17- and T_REG_-inducing cytokines, including IL-23 and transforming growth factor β (TGF-β) (Fig. 2e, f). Notably, IL-6 expression by PDA_TME_ DC occurred exclusively in TGF-β^+^ cells (Fig. 2f). Thus, PDA_TME_ DCs influence naive T cells to differentiate into IL-17A^+^IFNγ^+^ CD4^+^ T cells and are phenotypically equipped with the cytokine requirements for T_H_17-differentiation.

### CD4^+^ T-cell differentiation by PDA_TME_ DC is independent of TLR2 and Dectin-1 but is modulated by retinoic acid signaling

As DC cytokine production and capacity for T-cell polarization may be dependent on specific environmental cues, we interrogated PDA-associated DC subsets for expression of innate immune receptors linked to T_H_17 or T_REG_ induction. PDA_TME_ DC expressed elevated Dectin-1, CD206, and TLR2 cells compared with splenic DC populations, with the vast majority of Dectin-1^+^ PDA_TME_ DC co-expressing TLR2 and ICOSL (Supplementary Figure 3A–C). We have recently shown that the PDA TME is rife with Dectin-1 and TLR2 ligands^21,22^. Consistent with their higher Dectin-1 and TLR2 expression, PDA_TME_ DC secreted more TNFα, IL-6, and IL-10 than splenic DC in the presence of TLR2 (Pam_2_CSK_4_) and/or Dectin-1 (zymosan-depleted) ligands (Supplementary Figure 3D). To determine the influence of TLR2 and Dectin-1 signaling in PDA_TME_ DC modulation of CD4^+^ T-cell differentiation, we co-cultured Ova-restricted CD4^+^ T cells with Ova-pulsed PDA_TME_ DC in the presence or absence of zymosan-depleted or Pam_2_CSK_4_. Surprisingly, neither of these pattern recognition receptor ligands enhanced IL-17A expression in PDA_TME_ DC—CD4^+^ T-cell co-cultures (Supplementary Figure 3E). However, both Dectin-1 and TLR2 ligation in PDA_TME_ DC amplified their capacity to promote expression of IL-10, IL-6, IFN-γ, and TNFα in CD4^+^ T cells (Supplementary Figure 3E).

Activation of TLR2 and Dectin-1 can modulate tolerogenic or immunogenic T-cell responses by generating retinoic acid (RA) production in DC^23,24^. RA promotes the generation of FoxP3^+^ T_REGs_, while mitigating T_H_17 differentiation^12^. Of note, PDA_TME_ DC were the principal producers of RA among CD45^+^ tumor-infiltrating leukocytes and expressed higher RA than spleen DC (Supplementary Figure 4A and B). CD45^neg^ cells in primary PDA tumors, as well as in vitro cultured PDA tumor cells, also produced high RA (Supplementary Figure 4A–C). As T cells infiltrating PDA are presumably exposed to RA produced by both DC and the transformed epithelial cells, we investigated PDA_TME_ DC induction of T_H_ differentiation in the presence of exogenous *all-trans* RA (ATRA) or LE135, a RA receptor-α/β inhibitor. As expected, ATRA attenuated IL-17A and IFNγ secretion, upregulated IL-2 expression, and reduced ICOS surface expression on CD4^+^ T cells consistent with a phenotype of impaired IL-17^+^IFNγ^+^ cellular differentiation (Supplementary Figure 4D and E). By contrast, whereas LE135 did not have significant effects on IL-17 and IFNγ expression, it diminished FoxP3 expression on CD4^+^ T cells (Supplementary Figure 4F), suggesting a critical role of RA signaling in determining terminal T_H_-cell differentiation by PDA_TME_ DC.

### Distinct populations of PDA_TME_ DC have the requisite potential for inducing T_H_17 or T_REG_ differentiation

**Fig. 3:**
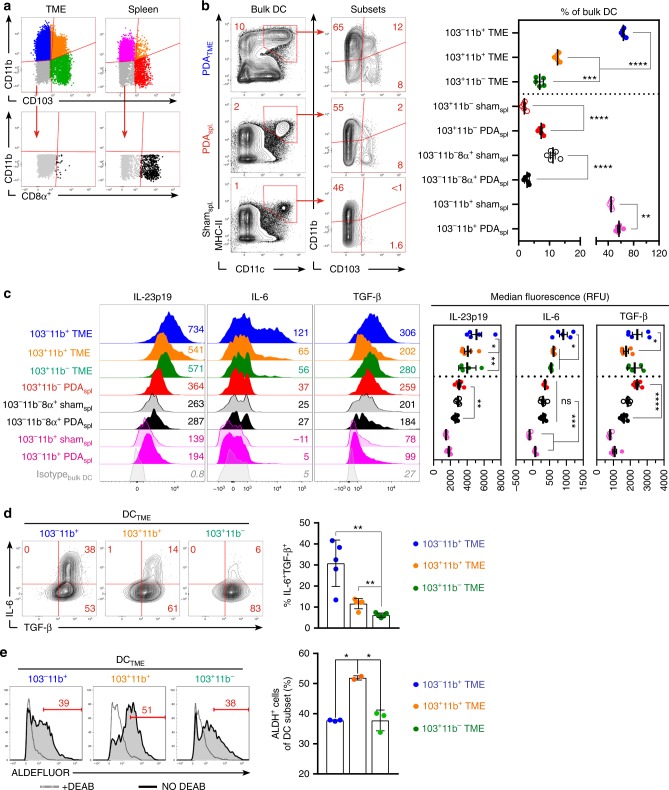
Specific subsets of PDA_TME_ dendritic cell (DC) exhibit distinct phenotypic properties. **a** The gating strategy for subset analysis of PDA_TME_ and spleen DC based on expression of CD11b, CD103, and CD8α is depicted. **b** Frequencies of PDA_TME_ and spleen DC subsets based on expression of CD11b, CD103, and CD8α were analyzed. Representative contour plots and quantitative results are shown. Experiments were repeated >5 times. **c** Histogram plots comparing cytokine expression in distinct DC subsets are shown. Numbers adjacent to histograms represent MFI ÷ 10. Quantitative data based on MFI are shown. Dotted-line separates PDA_TME_ DC from splenic DC controls. Experiments were repeated three times. **d** The frequency of IL-6 and TGF-β1 co-expression in PDA_TME_ DC subsets was tested. Representative contour plots and quantitative data are shown. This experiment was repeated three times. **e** Enzymatic activity of ALDH in PDA_TME_ DC subsets was determined by the ALDEFLUOR assay. Gates were determined by respective DEAB controls (dotted histogram) for each individual sample. This experiment was repeated three times (*p < 0.05, **p < 0.01, ***p < 0.001, ****p < 0.0001, *t*-test), SEM

Of note, tumor-associated macrophages did not express high CD11c in PDA (Fig. S4G and H). Divergent T-cell fates are directed by specific subpopulations of DC, which can be identified by the differential expression of CD11b and CD103 (Fig. 3a)^13,25^. The majority of DC in the PDA TME were CD103^−^CD11b^+^, followed by CD103^+^CD11b^+^, and CD103^+^CD11b^−^, respectively (Fig. 3b). Re-examination of DC cytokine production in the context of these subsets revealed that CD103^−^CD11b^+^ DC_TME_ produced greater IL-23p19, IL-6, and TGF-β, compared with other DC_TME_ populations (Fig. 3c). Similarly, CD103^−^CD11b^+^ DC_TME_ exceeded the other DC_TME_ subsets in co-expression of IL-6 and TGF-β (Fig. 3d). However, CD103^−^CD11b^+^ DC_TME_ produced lower RA than the CD103^+^CD11b^+^ DC_TME_ population (Fig. 3e).

### CD103^−^CD11b^+^PDA_TME_ DC induce the differentiation of immune-suppressive tumor-promoting FoxP3^*neg*^ CD4^+^ T cells

**Fig. 4:**
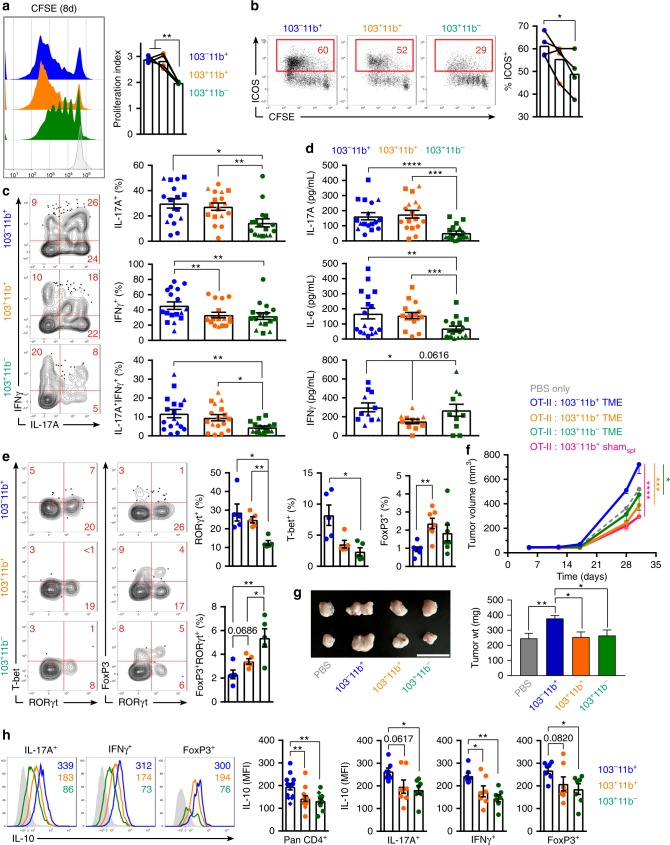
CD103^–^CD11b^+^PDA_TME_ dendritic cell (DC) promote the differentiation of suppressive FoxP3^*neg*^ CD4^+^ T cells. **a**–**e** Ova_323–339_ peptide-pulsed PDA_TME_ DC subsets were co-cultured with CFSE-labeled Ova-restricted CD4^+^ T cells for 5 days. **a** T-cell proliferation was determined by dilution of CFSE. Representative histograms and quantitative analysis of proliferation indices are shown. **b** T cells were analyzed for co-expression of CFSE and ICOS. Representative and quantitative data are shown. **c** CD4^+^ T cells were analyzed for co-expression of IFNγ and IL-17A. Representative contour plots and quantitative data are shown. Circles, squares, and triangles denote data points from individual mice, pooled from three independent experiments. **d** DC-CD4^+^ T-cell co-culture supernatant were analyzed for expression of IL-17A, IL-6, and IFNγ by cytometric bead array. **e** CD4^+^ T cells were analyzed for co-expression of T-bet/RORγt and FoxP3/RORγt. Representative contour plots and quantitative data are shown. Experiments were repeated three times with similar results. **f** KPC-derived tumor cells engineered to express Ovalbumin were implanted subcutaneously WT mice admixed with either phosphate-buffered saline (PBS) or Ova-restricted CD4^+^ T cells that had been entrained in vitro using each of the PDA_TME_ DC subsets or CD103^–^CD11b^+^ splenic DC pulsed with Ova_323–339_ peptide. Tumor volume was serially measured (*n* = 4–5 per group). **g** KPC-derived tumor cells were orthotopically implanted in pancreata of WT mice admixed with either PBS or each of the respective PDA_TME_ DC subsets that had been harvested from other pancreatic ductal adenocarcinoma (PDA) tumors. Representative gross images of intra-pancreatic tumors and quantitative data on tumor weight on day 25 are shown. This experiment was repeated twice (*n* = 5 per group; scale bars = 1 µm). **h** Ova_323–339_ peptide-pulsed PDA_TME_ DC subsets were co-cultured with CFSE-labeled Ova-restricted CD4^+^ T cells for 5 days. T cells stimulated with each respective DC subset were co-stained for IL-10 and either IL-17A, IFNγ, or FoxP3. Expression of IL-10 in pan-CD4^+^ T cells and in IL-17A^+^, IFNγ^+^, and FoxP3^+^ CD4^+^ T cells was determined. Representative histogram overlays and quantitative data are shown. This experiment was repeated three times (*p < 0.05, **p < 0.01, ***p < 0.001, ****p < 0.0001, *t*-test), SEM

To test whether the specific subsets of PDA_TME_ DC differentially influence CD4^+^ T-cell programming, we co-cultured the Ova-pulsed PDA_TME_ DC subsets with antigen-restricted CD4^+^ T cells. Both CD103^−^CD11b^+^ and CD103^+^CD11b^+^ DC_TME_ populations induced greater T-cell proliferation (Fig. 4a) and CD103^−^CD11b^+^ DC_TME_ induced higher ICOS expression than CD103^+^CD11b^−^ DC_TME_ (Fig. 4b). Further, the CD103^−^CD11b^+^ and CD103^+^ CD11b^+^ DC_TME_ populations induced the greatest fraction of IL-17A^+^ and IL-17F^+^ CD4^+^ T cells, including a substantial IL-17A^+^IFNγ^+^ population (Fig. 4c and Supplementary Figure 5A). Supernatant cytokine analysis confirmed that CD103^−^CD11b^+^ and CD103^+^CD11b^+^ DC_TME_ induce the highest expression of IL-17A and IL-6 in Ova-restricted CD4^+^ T-cell co-cultures (Fig. 4d). We next surveyed the transcriptional regulators in DC-entrained CD4^+^ T cells and similarly discovered that RORγt was expressed most robustly in CD4^+^ T cells that were primed by CD103^−^CD11b^+^ and CD103^+^CD11b^+^ DC_TME_ (Fig. 4e). However, the CD103^+^CD11b^+^ DC_TME_ induced a slightly higher percentage of FoxP3^+^ T cells, including a distinct population of FoxP3^+^RORγt^+^ cells, albiet at low frequency (Fig. 4e). The CD103^+^CD11b^−^ DC_TME_ population also promoted FoxP3^+^RORγt^+^ CD4^+^ T-cell differentiation. We questioned whether the FoxP3^+^ CD4^+^ T cells were able to also produce IL-17, as in previous reports^26^; however, FoxP3 expression was mutually exclusive of the production of IL-17F (Supplementary Figure 5A) or IL-17A (data not shown). Similarly, expression of IL-17A and FoxP3 in PDA-infiltrating CD4^+^ T cells in situ was also mutually exclusive, as was expression of IFNγ and FoxP3 (Supplementary Figure 5B). These data indicate that IL-17^+^ T cells in PDA are not bona fide FoxP3^+^ peripheral T_REGs_.

As CD103^−^CD11b^+^ DC_TME_ induced lower FoxP3 expression and higher IFN-γ, we hypothesized that CD4^+^ T cells primed by CD103^−^CD11b^+^ DC_TME_ would promote antitumor immunity and be less suppressive than T_H_ cells primed by the other PDA_TME_ DC subsets. To test this, we subcutaneously co-injected KPC tumor cells engineered to express ovalbumin with OT-II T cells entrained by the three distinct PDA_TME_ DC subsets. Surprisingly, mice receiving CD103^−^CD11b^+^ DC_TME_-primed OT-II T cells developed larger tumors than recipients of CD103^+^ CD11b^+^ DC_TME_-primed or CD103^+^CD11b^−^ DC_TME_-primed T cells (Fig. 4f). Similarly, direct transfer of CD103^−^CD11b^+^ DC_TME_ accelerated PDA growth in vivo whereas other DC_TME_ subsets did not modulate disease progression (Fig. 4g). Furthermore, antigen-restricted CD4^+^ T cells entrained by CD103^−^CD11b^+^DC_TME_ were inhibitory toward polyclonal CD8^+^ T-cell activation compared with CD4^+^T cells entrained by other DC_TME_ subsets (Supplementary Figure 5C). Polyclonal CD8^+^ T cells co-cultured with CD4^+^ T cells primed by CD103^−^CD11b^+^ DC_TME_ also exhibited increased cell death (Supplementary Figure 5D). Similarly, the supernatants that were conditioned by CD103^−^CD11b^+^ DC_TME_-CD4^+^ OTII T-cell co-cultures attenuated the proliferation and activation of polyclonal CD8^+^ and CD4^+^ T cells in vitro to a greater extent than the conditioned media of CD103^+^CD11b^+^ or CD103^+^CD11b^−^ DC_TME_-CD4^+^ OTII T-cell co-cultures (Supplementary Figure 5E). Moreover, T_H_ cells primed by CD103^−^CD11b^+^ DC_TME_ expressed greater IL-10 than those primed by other DC_TME_ subsets and this observation of higher IL-10 expression was constant despite gating on IL-17A^+^, IFNγ^+^, and FoxP3^+^ T cells (Fig. 4h). Further, blockade of IL-10 reversed the inhibitory effects of CD4^+^ T cells entrained by CD103^−^CD11b^+^ DC_TME_ (Supplementary Figure 5F). CD103^−^CD11b^+^ DC_TME_ transfer also failed to accelerate tumor growth in IL-10^−/−^ hosts (Supplementary Figure 5G). Collectively, these data indicate that CD103^−^CD11b^+^ DC_TME_ promote the differentiation of suppressive FoxP3^*neg*^ CD4^+^ T cells that express high IL-10 as well as substantial IL-17 and IFN-γ.

### CD103^−^CD11b^+^ PDA_TME_ DC induce a Tr1-like phenotype in CD4^+^ T cells

**Fig. 5:**
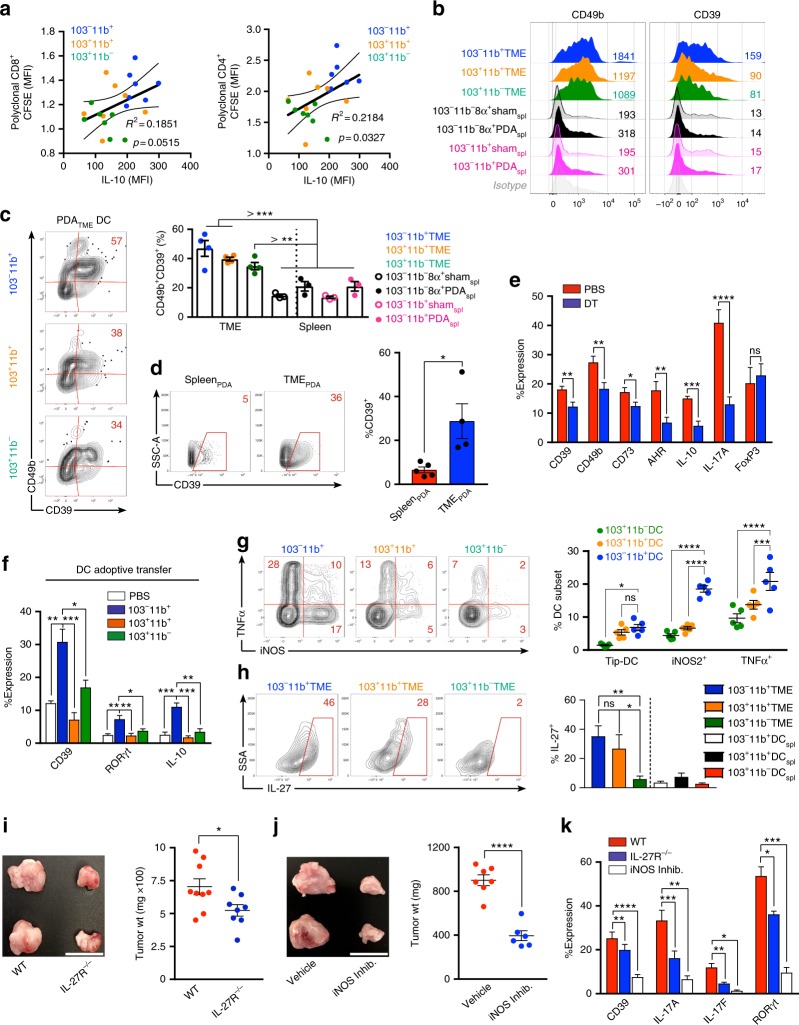
Pancreatic ductal adenocarcinoma (PDA)-infiltrating dendritic cell (DC) subsets direct distinct CD4^+^ T-cell programs in PDA. **a** CFSE-labeled naive CD8^+^ or CD4^+^ polyclonal T cells isolated from the spleens of WT mice were cultured for 96 h on αCD3ε/αCD28-coated plates in the presence of conditioned media from 8-day OT-II CD4^+^ T cell/PDA_TME_ DC subset co-cultures. Polyclonal T cells were assessed by flow cytometry for CFSE dilution. Each individual well from the OT-II/PDA_TME_ DC subset co-cultures was used to generate conditioned media for a single well of polyclonal CD8^+^ and CD4^+^ T cells. Scatter plots are shown correlating the CFSE dilution of polyclonal T cells to the IL-10 expression of OT-II T cells from the respective OT-II/PDA_TME_ DC subset co-culture used to generate conditioned media. Linear regression was used to determine the best-fit line (solid) and 95% confidence intervals (dotted lines); *p*-values indicate significance of a non-zero slope, determined by an *F*-test. This experiment was repeated twice. **b** Ova_323–339_ peptide-pulsed PDA_TME_ DC subsets and splenic DC subsets were co-cultured with Ova-restricted CD4^+^ T cells at a 1:5 ratio for 5 days. CD4^+^ T-cell expression of CD49b and CD39 were determined compared with isotype control. Representative histograms with MFIs are shown. This experiment was repeated >3 times (*n* = 3–6 mice). **c** Ova_323–339_ peptide-pulsed PDA_TME_ DC subsets and splenic DC subsets were co-cultured with Ova-restricted CD4^+^ T cells at a 1:5 ratio for 5 days. CD4^+^ T-cell co-expression of CD39 and CD49b were determined. Representative contour plots (PDA_TME_ DC) and quantitative data (PDA_TME_ DC, PDA_Spl._ DC, and Sham_Spl._ DC) are shown. Experiments were repeated >3 times (*n* = 3–6 mice). **d** Tumor and spleen were harvested on day 25 from mice bearing orthotopic PDA. Tumor-infiltrating and splenic CD4^+^ T cells were assessed for expression of CD39. Representative and quantitative data are shown. This experiment was repeated >5 times. **e** WT mice were made chimeric using bone marrow from CD11c.DTR mice. Animals were challenged with orthotopic PDA 7 weeks later. On day 12, mice began serial treatment with diphtheria toxin (DT) or phosphate-buffered saline (PBS) before sacrifice and tumor harvest on day 25. Intra-tumoral CD4^+^ T cells were gated on flow cytometry and tested for expression of CD39, CD49b, CD73, AHR, IL-10, IL-17A, and FoxP3. This experiment was repeated >5 times (*n* = 3–10 mice per group). **f** KPC-derived tumor cells were orthotopically implanted in pancreata of WT mice admixed with either PBS or each PDA_TME_ DC subset previously harvested from other PDA tumors. Tumors were then harvested on day 25 and intra-tumoral CD4^+^ T cells were gated on flow cytometry and tested for expression of CD39, RORγt, and IL-10. This experiment was repeated twice (*n* = 5 per group). **g** PDA_TME_ DC subsets were tested for co-expression of TNFα and iNOS. Representative contour plots and quantitative data are shown. This experiment was repeated three times. **h** PDA_TME_ and PDA_spl._ DC subsets were tested for expression of IL-27 compared with isotype control (data not shown). Representative contour plots and quantitative data are shown. This experiment was repeated >3 times (*n* = 5). **i** WT and IL-27R−/− mice were challenged with orthotopic PDA before sacrifice on day 25. Representative pictures of tumors and quantitative data of tumor weight are shown. Each dot represents data from a single mouse. This experiment was repeated three times (Scale bars = 1 µm). **j** Orthotopic PDA-bearing mice were serially treated with an iNOS inhibitor or vehicle. Tumor volume was measured at 25 days. Representative pictures of tumors and quantitative data of tumor weight are shown. Each dot represents data from a single mouse. This experiment was repeated twice (Scale bars = 1 µm). **k** WT and IL-27R−/− mice were challenged with orthotopic PDA before sacrifice on day 25. In addition, select cohorts of WT mice were treated with an iNOS inhibitor (*n* = 5 per group). CD4^+^ T-cell expression of CD39, IL-17A, IL-17F, and RORγt were determined. This experiment was repeated twice (*p < 0.05, **p < 0.01, ***p < 0.001, ****p < 0.0001, *t*-test), SEM

Revisiting data from the conditioned media assays, we observed that the frequency of FoxP3^+^ T_REGs_ in co-culture was associated with conditioned media that induced greater proliferation and activation of polyclonal CD4^+^ and CD8^+^ T cells (Supplementary Figure 5H). By contrast, higher IL-10 in CD4^+^ T-cell—PDA_TME_ DC co-cultures was associated with conditioned media that was more immunosuppressive (Fig. 5a). Tr1 cells are FoxP3^*neg*^ CD4^+^ T cells, which induce peripheral tolerance through diverse mechanisms^2,3^. They express IL-10 and IFNγ and have the capacity to produce IL-17. We postulated that CD4^+^ T cells primed by CD103^−^CD11b^+^ DC_TME_ induce characteristic Tr1 cells. Interestingly, all three subsets of PDA_TME_ DC, but not splenic DC, were able to induce expression of the integrin CD49b and the ecto-nucleosidase CD39, each associated with Tr1 cells (Fig. 5b). However, CD103^−^CD11b^+^ DC_TME_ induced the greatest co-expression of CD49b and CD39 (Fig. 5c). Accordingly, we found that a substantial portion of CD4^+^ T cells in the TME, but not the spleen, expressed CD39 in situ (Fig. 5d). Expression of CD49b, CD73, and the aryl hydrocarbon receptor (AHR) were also upregulated in situ in PDA-infiltrating CD4^+^ T cells compared with spleen (Supplementary Figure 5I). Expression of Tr1-associated markers in CD4^+^T cells in PDA was associated with high co-expression of Granzyme B (Supplementary Figure 5J). Moreover, DC depletion in vivo lowered tumor-infiltrating CD4^+^ T-cell expression of CD39, CD49b, CD73, AHR, IL-17, and IL-10 in PDA suggesting that, besides driving select cytokine expression, DC promote this Tr1-like surface phenotype in PDA-associated CD4^+^ T cells (Fig. 5e). Further, interrogation of the intra-tumoral T-cell phenotype in the DC_TME_ subset adoptive transfer experiments (Fig. 4g) suggested that CD103^−^CD11b^+^ DC_TME_ induce higher CD39, RORγt, and IL-10 expression than the other DC_TME_ subsets (Fig. 5f).

DC expression of inducible nitric oxide synthase (iNOS) has been implicated in the differentiation of suppressive FoxP3^*neg*^ Tr1 cells in inflammatory disease^27,28^. We found that of the PDA_TME_ DC populations, CD103^−^CD11b^+^ DC_TME_ produced the highest amounts of iNOS, followed by CD103^+^CD11b^+^ DC_TME_ (Fig. 5g). Notably, iNOS expression in DC was largely independent of TNFα production, indicating that PDA_TME_ DC are distinct from the TNFα^+^iNOS^+^ (Tip)-DC described in the context of infections and allergic diseases^29,30^. Tumor-associated macrophages also expressed significant iNOS in PDA (Supplementary Figure 5K). Besides iNOS, IL-27 has also been implicated in FoxP3^*neg*^ Tr1 cellular differentiation^31^. IL-27 was differentially upregulated in CD103^−^CD11b^+^ and CD103^+^CD11b^+^ PDA_TME_ DC subsets (Fig. 5h)_._ Other intra-tumoral leukocyte subsets only produced modest IL-27 in PDA (Supplementary Figure 5L). Moreover, both IL-27R deletion and iNOS inhibition were protective against PDA and, similar to DC depletion, reduced CD4^+^ T-cell expression of CD39, IL-17A, IL-17F, and RORγ in vivo (Figs. 5i–k). Notably, iNOS inhibition was not tumor protective in absence of DC (Supplementary Figure 5M). Collectively, these data suggest PDA_TME_ DC promote Tr1-like phenotypes in naive CD4^+^ T cells in an iNOS and IL-27 dependent manner.

### High expression of Tr1-associated genes correlates with poor prognosis in PDA patients

**Fig. 6:**
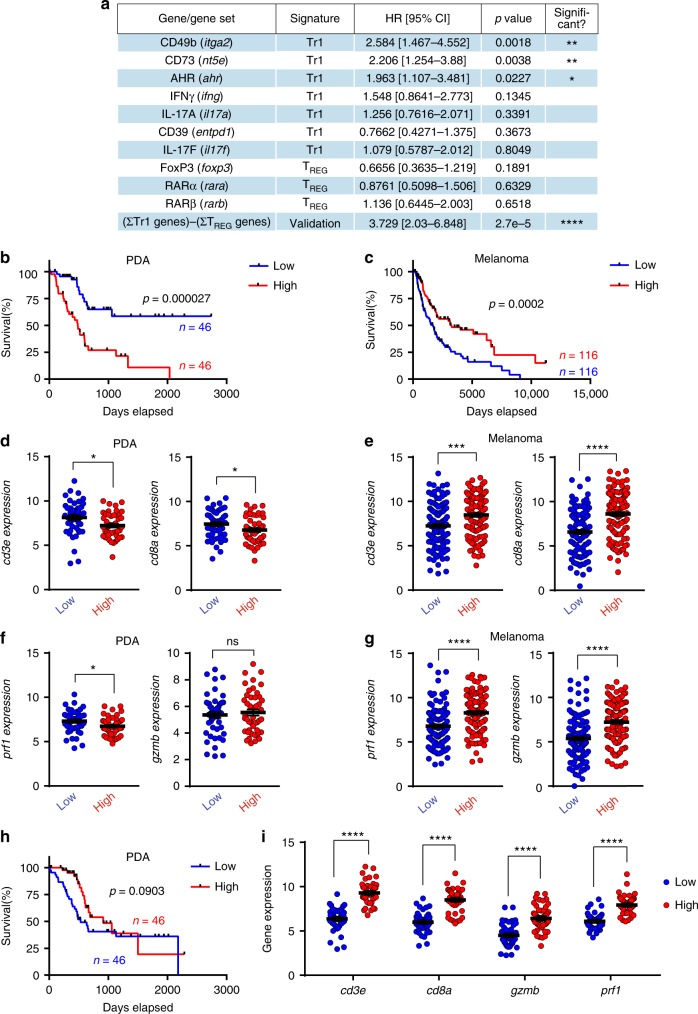
The suppressive program in CD4^+^ T cells blunting antitumor immunity in human pancreatic ductal adenocarcinoma (PDA) is distinct from other human cancers. **a** Data from PDA patients in the TCGA dataset were stratified on the basis of RNAseq gene expression (PolyA^+^ IlluminaHiSeq). Kaplan–Meier survival data comparing the highest and lowest patient quartiles (with the exception of *Il17a* and *Il17f*) are represented as HR with 95% confidence intervals, calculated using the log-rank (Mantel–Cox) method. Statistical comparisons for *Il17a* and *Il17f* represent patients with positive expression of each respective gene versus patients with zero expression. HR values <1 indicate increased OS, whereas values >1 indicate decreased OS for each gene. The developed “validation signature” indicates the gene expression sum of the 7 Tr1-associated genes subtracted by 3 Treg-associated genes (ΣTr1–ΣT_REG_). **b** Kaplan–Meier survival curve, plotting the highest (red) and lowest (blue) quartiles of PDA patients from the TCGA database stratified by expression of the ΣTr1–ΣT_REG_ signature, with tick marks indicating censored patients. *P*-value was determined using the log-rank (Mantel–Cox) method. **c** Kaplan–Meier survival curve, plotting the highest (red) and lowest (blue) quartiles of melanoma patients from the TCGA dataset, and analyzed with the same methods as in **b**. **d**, **e** The highest (red) and lowest (blue) quartiles of PDA (**d**) and melanoma (**e**) patients stratified by the ΣTr1–ΣT_REG_ validation signature were analyzed for *cd3e* and *cd8a* gene expression; *p*-values were determined by unpaired *t*-test with Welch’s correction. **f**, **g** Plots represent *prf1* and *gzmb* gene expression in the tumors of patients from the highest (red) and lowest (blue) quartiles of the ΣTr1–ΣT_REG_ validation signature from PDA patients (**f**) and melanoma patients (**g**), using RNAseq data from the TCGA database. All dot plots were analyzed with unpaired *t*-tests with Welch’s correction. **h** Kaplan–Meier survival curve, plotting the highest (red) and lowest (blue) quartiles of PDA patients from the TCGA database stratified by expression of three-gene T_REG_ signature, with tick marks indicating censored patients. *P*-value was determined using the log-rank (Mantel–Cox) method. **i** Plots represent *cd3e, cd8a*, *gzmb*, and *prf1* gene expression in the tumors of patients from the highest (red) and lowest (blue) quartiles of the three-gene T_REG_ signature from PDA patients. All dot plots were analyzed with unpaired *t-*tests with Welch’s correction (*p < 0.05, **p < 0.01, ***p < 0.001, ****p < 0.0001)

To validate our murine findings, we applied our knowledge of CD103^−^CD11b^+^ DC_TME_-induced T_H_ cells to patient data from The Cancer Genome Atlas (TCGA). By stratifying PDA patients on the basis of gene expression, we found that the highest quartile of patients expressing each of the “Tr1-related genes”—*CD49b*, *CD73*, and *AHR*—exhibited decreased overall survival (OS) (Fig. 6a). By contrast, expression of T_REG_-associated genes, including *FoxP3*, *RARα*, and *RARβ*, were not associated with survival differences (Fig. 6a). Given that the expression of *IFNγ* and *IL-17* were each mutually exclusive of FoxP3 expression (Supplementary Figure 5A and B), we designed a “validation signature” representing the sum of Tr1-related gene expression subtracted by the sum of T_REG_-related gene expression. This validation signature was significantly associated with a poor prognosis (Fig. 6a). Stratifying PDA patients by their validation signature scores into high and low quartiles, we discovered that patients in the lowest quartile had a profound OS advantage compared with patients in the highest quartile (Fig. 6b). Furthermore, log-rank (Mantel–Cox) survival curve comparisons between the highest and lowest quartiles displayed greater statistical significance than any gene individually, suggesting that the unique combination of genes in our validation signature may act collectively to drive outcome in PDA patients. This relationship between OS and the validation signature was not observed in other cancers, including breast carcinoma (BRCA), lung adenocarcinoma (LUAD), non-small cell lung cancer (NSLC), and head and neck squamous cell carcinoma (HNSCC) (Supplementary Figure 6A–D). Importantly, the survival phenotype in melanoma patients was opposite that of PDA patients, with patients in the highest validation signature quartile exhibiting increased OS compared with the lowest quartile (Fig. 6c). This may suggest that suppressive mechanisms blunting antitumor immunity in PDA are distinct from those found in other tumors. Interestingly, in low-grade gliomas (LGGs), a malignancy in which FoxP3^+^ T_REGS_ are not prognostically relevant^32^, OS based on our validation signature modeled PDA, albeit to a lesser degree of significance (Supplementary Figure 6E).

We next examined the differences in immune-related gene expression in PDA stratified by the ΣTr1–ΣT_REG_ validation signature score. Consistent with the OS data, the lowest quartile of PDA patients had greater transcriptional expression of *cd3e* and *cd8a*, suggesting the presence of more cytotoxic T cells in the tumor (Fig. 6d). By contrast, melanoma patients scoring high on the validation signature had greater T-cell infiltrates (Fig. 6e). Effective cytotoxic T-cell responses in the TME are mediated by granzymes and perforin, and have been shown to indicate cytolytic activity in tumors across the TCGA database^33^. We found that the high validation signature quartile of PDA patients expressed reduced *prf1* compared with the lowest quartile, whereas *gzmb* was similarly expressed in both groups (Fig. 6f). By contrast, melanoma (Fig. 6g) and BRCA (Supplementary Figure 6F) patients in the high validation signature quartile expressed significantly higher cytolytic markers. A similar pattern was seen when grouping all cancers in the TCGA database (Supplementary Figure 6G). Collectively, these data suggest that our ΣTr1–ΣT_REG_ signature is associated with less cytolytic activity in PDA, but greater cytolytic activity in other cancers.

These results made us question the relationship between T_REG_-associated gene expression and OS in PDA patients. Because Tr1-related genes in the validation signature may also be expressed on T_REGS_, we created another signature using genes associated exclusively with T_REGS_. Sorting PDA patients by a simple three-gene signature representing the sum of *foxp3*, *rara*, and *rarb* expression, we discovered that the highest quartile of scorers conferred a trend towards greater OS than the lowest quartile (Fig. 6h). Moreover, the highest quartile of PDA T_REG_ signature scorers exhibited significantly greater expression of *cd3e*, *cd8a*, *gzmb*, and *prf1* than the PDA patients of the lower quartile (Fig. 6i), suggesting that intra-tumoral T_REGS_ are accompanied by concomitant infiltrates of cytotoxic T cells in PDA patients. Collectively, these analyses validate our preclinical identification of immunosuppressive FoxP3^*neg*^ CD4^+^ T cells induced by CD103^−^CD11b^+^ PDA_TME_ DC. Furthermore, implications of the differentiation phenotype of these T_H_ cells are translationally mirrored by the lower OS and diminished intra-tumoral cytolytic activity of PDA patients expressing allied genes.

## Discussion

PDA is a devastating diagnosis and carries a grim prognosis. Little progress has been made in the development of new treatments and clinical trials testing checkpoint-based immunotherapies have yielded disappointing results^5^. Nevertheless, there is rationale for pursuing immunotherapeutic manipulation of this disease as immunity or its subversion are strongly associated with disease progression in PDA^34^. However, the drivers of CD4^+^ T-cell differentiation in the PDA TME and the character of the suppressive T_H_ phenotype remain incompletely understood.

Tumor-infiltrating CD8^+^ T cells in melanoma are directed toward CTL function by CD103^+^ DC in mice and CD141^+^ DC in humans^25^. These DC subsets are responsible for trafficking tumor antigen leading to both direct CD8^+^ T-cell stimulation and antigen hand-off to resident myeloid cells. These effects required CCR7. However, unlike melanoma, CD8^+^ T cells are largely irrelevant in PDA as their deletion or depletion does not accelerate oncogenesis^18,19^. Accordingly, we found that PDA_TME_ DC do not differentially affect CD8^+^ T-cell activation; conversely, PDA_TME_ DC have distinctive influences on T_H_ cell programming.

Our cellular ablation experiments using CD11c.DTR mice suggested that, as a bulk population, DC are tumor permissive in PDA in a CD4^+^ T-cell-dependent manner. The CD11c.DTR model has been described as being specific to DC and is the most widely used method of DC depletion^35,36^. Nevertheless, this model has limitations relating to both the cellular plasticity of DC and to the recent observation that it is associated with neutrophilia upon DTR administration^37^. Collectively, we show that PDA_TME_ DC express high T_H_1-, T_REG_-, and T_H_17-inducing cytokines and distinctly co-express select inflammatory mediators including IL-12/IL-23 and IL-6/TGF-β. PDA_TME_ DC capture antigen with marked avidity and are highly mature, including expressing elevated CCR7 and ICOSL. However, PDA_TME_ DC phenotype and functional capacity for driving T_H_ cell differentiation is dictated by their classification based on CD103 and CD11b expression. Our data suggest that CD103^−^CD11b^+^ DC_TME_ are a major driver of T_H_17-inducing cytokines; however, CD103^+^CD11b^−^ DC make equivalent levels of TGF-β to CD103^−^CD11b^+^ DC, and other DC subsets also sufficiently produce additional cytokines that may contribute substantially to Th17 polarization. The CD103^+^CD11b^−^ subset is least abundant and is inefficient at inducing T_H_ cell proliferation or expression of costimulatory ligands, but induces high FoxP3 expression and FoxP3/RORγt co-expression. The CD103^+^CD11b^+^ subset also expresses low levels of inflammatory and regulatory cytokines, but produces high ATRA. This subset also induces high FoxP3^+^ T_REGs_ and has reduced capacity for T_H_1 differentiation. However, despite the ostensible tolerogenic T_H_ phenotype induced by the CD103^+^CD11b^−^ and CD103^+^CD11b^+^ DC populations, CD4^+^ T cells entrained by these DC subsets do not have tumor-promoting or immune-suppressive function based on direct DC adoptive transfer experiments, T_H_-cell transfer experiments after DC entrainment, and in vitro conditioned media experiments. By contrast, the CD11b^+^CD103^−^ population, which is the most abundant PDA_TME_ DC subset, have striking tumor-promoting properties based on both direct DC adoptive transfer and transfer of DC-entrained T_H_ cells. This subset expresses high levels of inflammatory and regulatory cytokines, low ATRA, and induces high CD4^+^ T-cell proliferation and expression of costimulatory molecules. Even more surprising considering their tumor-permissive properties, the CD103^−^CD11b^+^ DC subset induces CD4^+^ T cells to express high T_H_1- and T_H_17-family cytokines and transcription factors, including a substantial IFNγ^+^IL17^+^ population, which co-expresses IL-10. In vitro co-culture and in vivo adoptive transfer experiments suggest that the CD103^−^CD11b^+^ subset drives Tr1-associated surface marker expression in CD4^+^ T cells. We found that the immune-suppressive and tumor-promoting effects of these T_H_ cells are linked, at least in part, to their expression of IL-10.

Tr1 cells have been investigated in allergic and inflammatory contexts and in infectious disease and express high CD39, CD73, CD49b, and AHR^38^. In these settings, Tr1 cells prevent excessive inflammatory responses, which can lead to autoimmunity, maintaining comparative immune tolerance. However, the role of Tr1 cells in cancer is not well studied. Consistent with our data, the suppressive function of Tr1 cells is mediated in part by their production of IL-10, which dampens the function of both APCs and antigen-specific effector T cells^3^. Tr1 cells are by definition FoxP3^*neg*^ and reportedly produce modest levels of IFNγ; however, these cells can also express robust IL-17 as we demonstrate. This is particularly notable in PDA as transformed pancreatic ductal epithelial cells upregulate IL-17R and its ligation is mitogenic^14^. Tr1 cells can kill both CD8^+^ T cells and myeloid cells through the perforin-granzyme B pathway^38^. We found that T_H_ cells in PDA that express the characteristic Tr1 phenotype express high granzyme B. Accordingly, in human PDA low expression of our Tr1-associated validation signature was accompanied by a higher CD8^+^ T-cell infiltrate and a more immunogenic CD8^+^ T-cell profile. Besides targeting CD8^+^ T cells, Tr1 cells can also suppress innate immunity by inhibiting the NLRP3 inflammasome in myeloid cells in an IL-10-dependent mechanism^39^. Of note, we previously reported that NLRP3 signaling has tolerogenic effects in PDA^40^. However, we do not have direct evidence that Tr1 cells inhibit NLRP3 in PDA. Further, we previously showed that the T-cell mediated suppressive influence on other T-cell subsets can be profound compared with interface between myelomonocytic cells and T cells because of intimate proximity between T cells subsets in the TME^18^.

We elucidate biochemical factors governing regulation of T_H_ phenotype by DC in PDA. Notably, whereas Dectin-1 and TLR2 signaling differentially affected PDA_TME_ DC capacity to secrete cytokines, it did not appreciably alter their capacity for CD4^+^ T-cell differentiation. By contrast, ATRA signaling in PDA_TME_ DC modulated the balance between IL-17-producing T_H_ cells and T_REGs_. However, the distinctive Tr1-like T_H_ differentiation noted in PDA was driven by DC expression of IL-27 and iNOS. We show that all subsets of DC_TME_ produce iNOS; however, CD103^−^CD11b^+^ DC are the highest expressers. Moreover, akin to depleting DC, targeting IL-27 and iNOS were tumor protective in PDA and abrogated many elements of the characteristic CD4^+^ T-cell Tr1-like surface phenotype and cytokine profile in the TME, although mechanistic links to specific DCs subsest remains to be defined in vivo. Each of these approaches may be attractive strategies for testing in experimental immunotherapy regimens in PDA. Tr1 cells have been characterized in HNSCC, Hodgkin’s lymphoma, hepatocellular carcinoma (HCC) and colorectal cancer (CRC)^41^. Generation of Tr1 cells from naive CD4^+^ T-cell precursors is promoted primarily via immature DC in models of HNSCC, HCC, and liver metastases of CRC^42,43^. In CRC metastases, Tr1 cells comprise ~30% of the tumor-infiltrating lymphocytes and expressed a diversity of cytokines^44^. Moreover, Tr1 cells in CRC exhibited an in vitro suppressive activity ~50 times more potent than FoxP3^+^ Tregs. Nevertheless, the clinical impact or consequences of Tr1 cell expansion in cancer is still uncertain. Further, there is no clear direct link between Tr1 cell, the DC subsets we investigated, and patient outcomes in PDA.

Surprisingly, our human data show that FoxP3 expression or upregulation of a three-gene T_REG_ signature was not associated with reduced survival in PDA. This contrasts with a recent report suggesting that targeting T_REGs_ is tumor protective in preclinical models of PDA^45^. Of note, T_REGs_ can have secondary tolerogenic influences in PDA and other malignancies by reciprocally suppressing DC expression of costimulatory ligands^45,46^. However, our analysis of TCGA data suggests that intra-tumoral T_REGs_ are accompanied by concomitant infiltrates of highly activated cytotoxic T cells in PDA patients, which may account for the absence of a negative prognosis associated with FoxP3 or related gene expression in human disease. Of note, the prognostic value of FoxP3^+^ Tregs in cancer is controversial. A recent meta-analysis of 76 studies encompassing 17 types of cancer, including 15,512 cancer cases, suggested that in aggregate FoxP3^+^ Tregs had a negative effect on survival^47^. However, the prognostic effect varied greatly according to tumor type. High FoxP3^+^ Tregs infiltration was significantly associated with shorter survival in the most solid tumors, including cervical, renal, melanomas, and breast cancers, whereas FoxP3^+^ Tregs were associated with improved survival in colorectal, head and neck, and esophageal cancers.

Immunotherapy has made impressive inroads in clinical therapy for several cancers including melanoma, NSLC, renal cell carcinoma, and HCC. However, PDA has been recalcitrant to checkpoint-based immunotherapy^5^. T-cell scarcity and chemokine-mediated exclusion of T cells from the tumor milieu, reduced T-cell expression of checkpoint receptors, and inability of therapeutic mAbs to physically access PDA-related peri-tumoral fibrosis have each been purported as potential mechanisms of therapeutic resistance in PDA^21,48,49^. Our current work may further illuminate this conundrum. We found that the suppressive phenotype of CD4^+^ T cells in PDA are distinct compared with other cancers. Specifically, the ΣTr1–ΣT_REG_ signature is associated with a markedly poor prognosis and immune suppression in PDA, whereas the same signature is associated with tumor protection and antitumor immunity in melanoma. Collectively, our data suggest that, as a result of a distinctive DC-T_H_ axis, PDA may harbor an immune environment that is different from many other cancers such as melanoma, suggesting that PDA requires a more tailored approach to immunotherapy.

## Methods

### Animals

C57Bl/6J (H-2K_b_), Balb/c (H-2K_d_), CD11c^GFP^.DTR, B6N.129P2-*Il27ra*^*tm1Mak*^/J, B6.129P2-*Il10*^*tm1Cgn*^/J, B6N.129P2-*Il27ra*^*tm1Mak*^/J, CD45.1, OT-I, and OT-II mice were purchased from the Jackson Laboratory. KPC mice^50^ were a gift from M. Phillips (New York University). Animals were housed in a clean vivarium and fed standard mouse chow. Bone marrow chimeric animals were created by irradiating mice in a SAARP irradiator (xStrahl Life Sciences). Mice were exposed to two fractions of irradiation (550 rads per dose) interspaced by 6 h, followed by the intravenous (i.v.) bone marrow transfer (10^7^ cells) of nonirradiated donors 12 h after the last dose of radiation. Chimeric mice were used in experiments 6–8 weeks later. To deplete DC in chimeric mice, 1 µg diphtheria toxin was administered intraperitoneal (i.p.); with subsequent doses of 0.2 µg q48h. In select experiments, CD4^+^ (GK1.5) or CD8^+^ T cells (53–6.72; each 150 µg, i.p., q72h; BioXcell) were depleted with neutralizing mAbs as we have previously described^19^. To inhibit iNOS, mice were serially treated with 1400 W dihydrochloride (50 µg, q48h, Sigma-Aldrich). Animal procedures were approved by the New York University School of Medicine Institutional Animal Care and Use Committee.

### Tumor experiments

The “FC1242” (C57BL/6 background) murine PDA cell line was isolated from the pancreatic tumor of a KPC mouse (*pdx1*^*cre/+*^;*KRas*^*LSL-G12D*^/^+^; *p53*^*R172H/+*^) as we described previously^51^. In select experiments, we utilized FC1242 KPC-derived tumor cells (1 × 10^6^), which we engineered to express OVA using pCI-neo-cOVA (gift of Maria Castro; Addgene plasmid # 25097) as we described^21^. To establish orthotopic pancreatic lesions in vivo, tumor cells (10^5^) were suspended in phosphate-buffered saline (PBS) with Matrigel (BD) in a 1:1 ratio and were injected into the body of the pancreas via laparotomy. In select experiments, DC (2.5 × 10^4^) were admixed with tumor cells and co-transferred to pancreata of mice. In other experiments, OTII T cells (5 × 10^4^) were admixed with equal numbers of FC1242 and FC1242.Ova tumor cells (2.5 × 10^4^ each) and administered subcutaneously. For our liver metastases model, PDA cells (1 × 10^6^) were administered into the portal venous system via direct splenic injection followed by splenectomy, as we reported^52^.

### Cell preparation and flow cytometry

Single-cell suspensions for flow cytometry were prepared as we described previously^18,19^. Briefly, PDA tumors were placed in cold RPMI-1640 with Collagenase IV (1 mg/mL; Worthington Biochemical), Trypsin inhibitor from soybean (100 µg/mL; Sigma), and DNase I (2 U/mL; Promega) and minced with scissors to sub-millimeter pieces. Tissues were then incubated at 37 °C for 30 min with gentle shaking every 5 min. Specimens were passed through a 70 μm mesh, and centrifuged at 350 *g* for 5 min. The cell pellet was resuspended in cold PBS with 1% fetal bovine serum . Cell labeling was performed after blocking FcγRIII/II with an anti-CD16/CD32 mAb (eBioscience) by incubating 1 × 10^6^ cells with 1 μg of fluorescence conjugated mAbs directed against murine CD44 (IM7), CD206 (C068C2), CD3 (17A2), CD4 (RM4-5), CD8 (53–6.7), CD39 (Duha5), CD45 (30-F11), CD49b (DX5), CD73 (TY/11.8), CD11b (M1/70), CD11c (N418), CD103 (2E7), MHC II (M5/114.15.2), IL-2 (5H4), IL-4 (11B11), IL-6 (MP5-20F3), IL-10 (JES5-16E3), IL-17A (TC11-18H10.1), IL-17F (8F5.1A9), IL-27 (MM27-7B1), CD68 (FA-11), F4/80 (BM8), IFN-γ (XMG1.2), TNFα (MP6-XT22), ICOS (15F9), CD62L (MEL-14), IL-23p19 (fc23cpg), IL-12/IL-23p40 (C11.5), IL-12p35 (C15.6), RORγt (AFKJS-9), AHR (4MEJJ), TLR-2 (CB225), TGF-β (TW7-16B4), ICOSL (HK5.3), Ms IgG (Poly4053), Rt IgG (Poly4054; all Biolegend), Tbet (eBio4B10), iNOS (CXNFT), IL-13 (eBio13A), FoxP3 (FJK-16s; all ebiosciences), and Dectin-1 (2A11, Abcam). Dead cells were excluded from analysis using zombie yellow (Biolegend). Aldefluor assays were performed using ALDEFLUOR kit and DEAB Reagent as per the manufacturer’s protocol (Stemcell Technologies). Intracellular cytokine staining was performed after 3–5 h in vitro stimulation with eBioscience Cell Stimulation Cocktail (plus protein transport inhibitors) using the Foxp3 Transcription Factor Staining Buffer Kit (eBioscience) for cytokines and transcription factors. Flow cytometry was carried out on the LSR-II flow cytometer (BD Biosciences). Data were analyzed using FlowJo v.10.1 (Treestar). DC antigen capture experiments were performed using FITC-Albumin (Sigma) as previously described by us^53^. Supernatant cytokine levels were measured in a cytometric bead array as per the manufacturer’s protocol (BD Biosciences).

### In vivo T-cell proliferation and differentiation assay

The spleens of OT-II and OT-I mice were separately minced with a razor and mashed through a 40 μm strainer to form a single-cell suspension. Erythrocytes were hypotonically lysed with an ammonium chloride-based red blood cell (RBC) lysis buffer and the appropriate T cells were isolated with CD4^+^ T-cell (for OT-II) and CD8^+^ T-cell (for OT-I) isolation kits (Miltenyi). Purified CD4^+^ OT-II and CD8^+^ OT-I T cells were counted, combined at a 1:1 ratio, and stained with 5 μM CellTrace™ CFSE (Life Technologies) for 5 min at room temperature, as previously outlined^54^. CD45.1 recipient mice were injected retro-orbitally (RO) with the Ova-specific T-cell mixture (8 × 10^6^ to 1 × 10^7^) suspended in saline. After 36 h, pancreatic tumors and spleens of tumor-bearing mice were enzymatically dissociated into single-cell suspensions and incubated with both Ova_257–264_ and Ova_323–339_ peptides (10 μg/mL; InvivoGen) at 37 ˚C for 1.5 h. Cellular suspensions were washed and CD11c^+^ cells were magnetically enriched with ultrapure CD11c microbeads as per the manufacturer’s protocol (Miltenyi). Peptide-pulsed DC (2 × 10^5^) were injected RO into the CD45.1 recipients, which were euthanized 6 days post-DC inoculation. Recipient spleens and mesenteric lymph nodes were harvested for flow cytometric analysis.

### In vitro DC–T-cell co-culture assays

Tumor-bearing and control mice were euthanized and pancreatic tumors and spleens were dissociated into single-cell suspensions, pulsed with Ova_323–339_ (10 μg/mL), and magnetically enriched for CD11c^+^ cells, which were then co-cultured at a 1:5 ratio with OT-II T cells (5 × 10^5^) in a 96-well U-bottom plate. Alternatively, antigen-pulsed DC subsets were FAC-sorted on the BD FACSAria™ II using the 100 μm nozzle, centrifuged at 350 × *g*, and plated onto a 96-well V-bottom plate with 25,000 purified OT-II CD4^+^ T cells at a 1:5 DC:T-cell ratio. After 7–8 days, supernatant was collected and cells were harvested for downstream analysis. T cells were labeled with CFSE in some experiments as per the protocol outlined by Quah et al.^54^. For select experiments, co-cultures occurred in the presence of ATRA (1 μM), LE135 (1 μM, both Sigma), Zymosan depleted (100 µg/ml, InvivoGen), or Pam_2_CSK_4_ (10 ng/ml, InvivoGen). After 5–8 days, supernatant was collected and cells were harvested for analysis. Allogeneic T-cell proliferation in an MLR was performed as we previously described^53^.

### Conditioned media suppression assay

Polyclonal CD4^+^ and CD8^+^ T cells from the spleens of WT mice were isolated using the CD4^+^ T-cell and CD8^+^ T-cell isolation kits (Miltenyi), respectively, combined at a 1:1 ratio, labeled with CFSE, and 100,000 T cells from this mixture were plated in 50 µL of complete assay media onto an anti-CD3ε mAb-coated 96-well flat-bottom plate. Next, 50 μL of the 200 μL supernatant from the DC subset/OT-II co-cultures was added to the polyclonal T cells and supplemented with 1 μg/mL of soluble anti-CD28. After 72–84 h, T cells were harvested and assessed by flow cytometry for CFSE dilution and T-cell activation. In select experiments, a neutralizing IL-10 mAb was added (1 μg/ml, Biolegend).

### T-cell adoptive transfer experiments

After 8 days, differentiated OT-II CD4^+^ T cells from the DC subset co-cultures were harvested and fluorescence-activated cell (FAC)-sorted. In total, 5000 purified OT-II T cells were then subcutaneously co-injected with a 1:1 mix of 50,000 FC1242 cells and 50,000 Ova-expressing FC1242 cells. For DC subset co-cultures that did not have extensive OT-II T-cell proliferation (e.g., CD103^+^ DC_TME_), we pooled OT-II T cells sorted from multiple co-culture wells to be able to harvest enough T cells for injection. Tumor volume was measured by calipers as previously described^53^. Because of low cell numbers in the subcutaneous tumor cell injections, some tumors did not seed and were excluded from analysis if there was no post-implant growth throughout the 30 days.

### TCGA bioinformatics and survival data

RNA sequencing data (polyA^+^ IlluminaHiSeq) representing genome-wide mRNA from patient tumor samples was obtained from individual datasets downloaded from the University of California Santa Cruz Xena browser (UCSC Xena, http://xena.ucsc.edu). PDA (*n* = 182), BRCA (*n* = 1200), melanoma (SKCM, *n* = 462), HNSCC (*n* = 562), lung cancer (LUNG (denoted as “NSLC”), *n* = 1109), LUAD (*n* = 564), LGG (*n* = 525), and TCGA PanCancer (*n* = 10,264; data from the Cancer Genome Atlas Research Network; https://www.cancer.gov/tcga) datasets were downloaded (08/2017) and filtered to exclude samples with incomplete clinical data (i.e., non-zero, non-“NA”). The validation signature was calculated as the sum of “Tr1 signature” genes subtracted by the sum of “Treg signature” genes as depicted in Fig. 6, and patients from each respective dataset were ranked by validation signature score. Each respective dataset was then divided into quartiles and the highest and lowest quartiles were compared. Kaplan–Meier plots were generated using GraphPad Prism software, and statistical significance of survival curves was determined using the log-rank (Mantel–Cox) test. Hazard ratios of the highest and lowest quartiles of the Pancreatic Adenocarcinoma (PAAD) dataset were reported using the log-rank method, also calculated in GraphPad Prism’s survival analysis statistics. mRNA transcript expression of *CD3e*, *CD8a*, *Gzmb*, and *Prf1* was compared between the highest and lowest quartiles of the specified TCGA patients stratified by indicated signatures, and significance was determined by unpaired two-tailed *t-*tests with Welch’s correction. For in vitro assays and mouse tumor experiments, data are presented as mean ± standard error. Statistical significance was determined by the two-tailed Student’s *t*-test with Welch’s correction; paired and unpaired analysis were conducted where appropriate and indicated in the figure legends.

### Ethical statement

All animal experiments were conducted in accordance with a protocol approved by the New York University Institutional Animal Care and Use Committee (IACUC).

### Reporting summary

Further information on experimental design is available in the Nature Research Reporting Summary linked to this article.

## Supplementary information

Supplementary Information

Reporting Summary

## Data Availability

The data that support the findings of this study are available from the corresponding author upon reasonable request.
