# Supplementary material for: Specialized dendritic cells induce tumor-promoting IL-10+IL-17+ FoxP3neg regulatory CD4+ T cells in pancreatic carcinoma
Source: Nat Commun. 2019 Mar 29;10:1424. doi: 10.1038/s41467-019-09416-2 (PMC6441038; doi:10.1038/s41467-019-09416-2)
Supplement: Supplementary file 1 — Supplementary Information [file 41467_2019_9416_MOESM1_ESM.pdf]

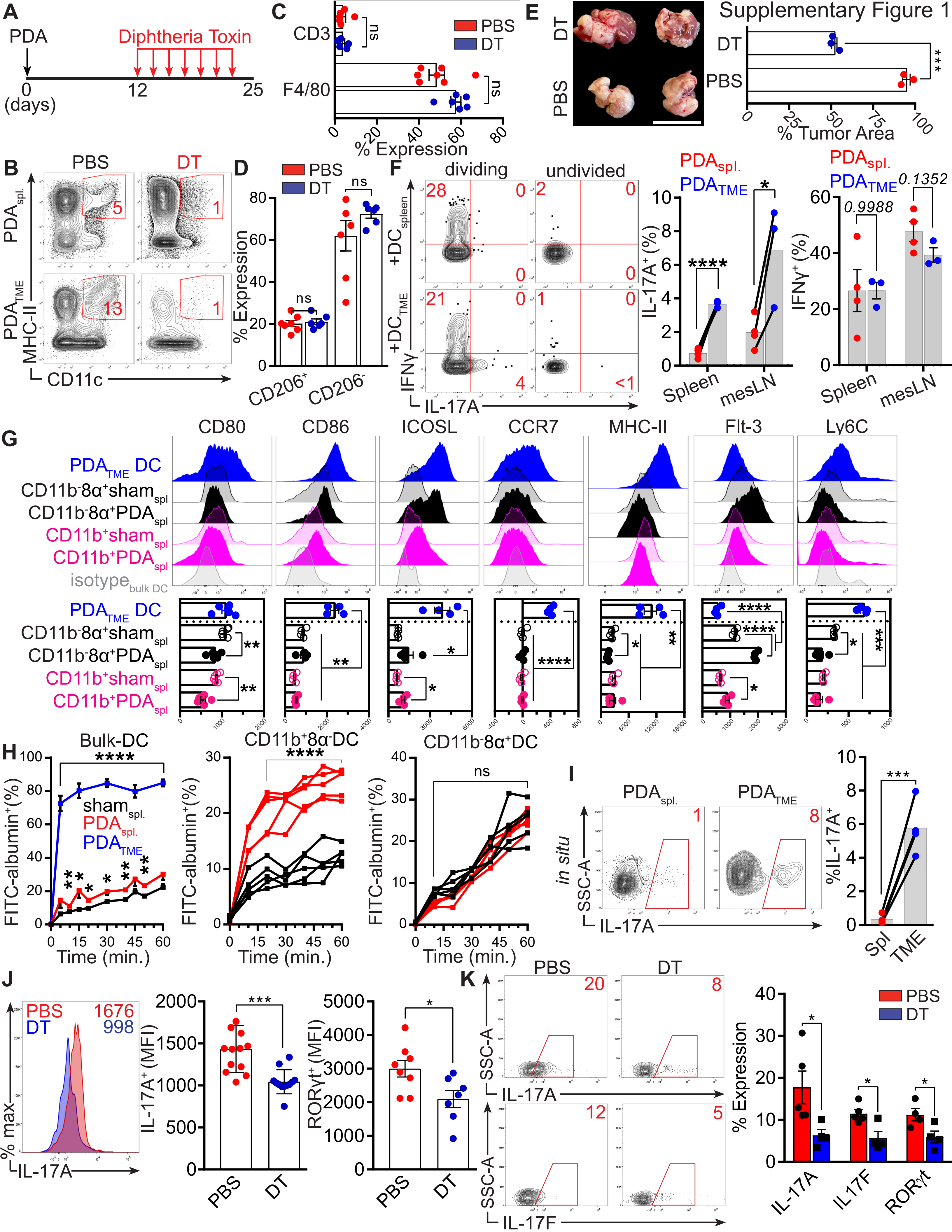
Supplementary Figure 1
A
PDA
Diphtheria Toxin
0 (days)
12
25
C
CD3
F4/80
PBS
DT
ns
% Expression
E
DT
PBS
% Tumor Area
***
B
PBS
DT
PDAspl.
PDATME
MHC-II
CD11c
D
CD206+
CD206-
% Expression
F
dividing
undivided
+DCspleen
+DCTME
IFNγ
IL-17A
PDAspl.
PDATME
IL-17A+ (%)
IFNγ+ (%)
Spleen
mesLN
0.9988
0.1352
G
CD80
CD86
ICOSL
CCR7
MHC-II
Flt-3
Ly6C
PDATME DC
CD11b-8α+shamspl
CD11b-8α+PDAspl
CD11b+shamspl
CD11b+PDAspl
isotypebulk DC
H
Bulk-DC
CD11b+8α-DC
CD11b-8α+DC
FITC-albumin+(%)
Time (min.)
shamspl.
PDAspl.
PDATME
ns
I
in situ
SSC-A
IL-17A
PDAspl.
PDATME
%IL-17A+
Spl
TME
J
PBS 1676
DT 998
% max
IL-17A
IL-17A+ (MFI)
RORγt+ (MFI)
PBS
DT
K
PBS
DT
SSC-A
IL-17A
IL-17F
% Expression
IL-17A
IL17F
RORγt

Supplemental Figure 1. PDA$_{TME}$ DC possess a distinctive phenotype.

(A) Schematic showing experiment assessing effects of DC depletion on PDA growth. CD11c.DTR bone marrow chimeric mice were administered orthotopic PDA. DT or vehicle were serially administered beginning on day 12 after tumor establishment. Mice were sacrificed on day 25.

(B) Ablation of DC populations in CD11c.DTR chimeric mice treated with DT was confirmed by flow cytometry analysis of co-expression of CD11c and MHC II. Representative flow cytometry contour plots are shown. This experiment was repeated more than 5 times.

(C) CD11c.DTR bone marrow chimeric mice were administered orthotopic PDA. DT or PBS were serially administered beginning on day 12 after tumor establishment. Mice were sacrificed on day 25 and tumor-infiltrating CD45$^+$ leukocytes were tested for expression of CD3 and F4/80. This experiment was repeated more than 3 times (n=5/group).

(D) CD11c.DTR bone marrow chimeric mice were administered orthotopic PDA. DT or PBS were serially administered beginning on day 12 after tumor establishment. Mice were sacrificed on day 25 and tumor-infiltrating F4/80$^+$ macrophages were tested for expression of CD206. This experiment was repeated twice (n=5/group).

(E) CD11c.DTR bone marrow chimeric mice were administered PDA tumor cells via a single splenic injection followed by splenectomy. DT or PBS were serially administered beginning on day 12 after tumor establishment. Mice were sacrificed on day 25. Representative images of livers and quantitative analysis of percentage of liver surface area replaced by tumor are shown. This experiment was repeated twice (n=3/group; scale bars = 1μm).

(F) PDA$_{TME}$ and PDA$_{spl.}$ DC were harvested, loaded in equal number with Ova$_{323-339}$ or Ova$_{257-264}$ peptide and administered i.p. to cohorts of CD45.1 mice that had been transferred i.v. 2 days

prior with equal numbers of CFSE-labeled OT-I and OT-II T cells. On day 7 after PDA_{TME} or PDA_{spl} DC.Ova administration, spleen and mesenteric lymph nodes (LN) were harvested and $CD45.2^+$ $CD4^+$ antigen restricted T cells tested for co-expression of IFNγ and IL-17A. Representative contour plots and quantitative data comparing priming using PDA_{TME} or $PDA_{spl.}$ DC are shown. This experiment was repeated twice.

(G) Comparison of PDA_{TME}, $PDA_{spl.}$, and $sham_{spl}$ DC subsets expression of the indicated surface markers. Representative histograms and quantitative data based on MFI are shown. Gating strategy for each marker was determined by the fluorescence of the respective isotype control (not shown). This experiment was repeated more than 3 times.

(H) PDA_{TME}, $PDA_{spl.}$, and $sham_{spl}$ DC from PDA-bearing or control mice were isolated and pulsed for varying durations with FITC-labeled albumin. Bulk-DC (first panel) as well as splenic $CD11b^+CD8\alpha^-$ DC (middle panel), and $CD8\alpha^+CD11b^-$ DC (last panel) subsets were analyzed by flow cytometry for albumin uptake. This experiment was repeated 3 times and performed in replicates of 5.

(I) Tumor-infiltrating and spleen $CD4^+$ T cells were harvested on day 25 from mice bearing orthotopic PDA and tested for expression of IL-17A. Representative contour plots and quantitative data are shown. Experiments were repeated more than 5 times.

(J) CD11c.DTR bone marrow chimeric mice were administered orthotopic PDA. DT or PBS were serially administered beginning on day 12 after tumor establishment. Mice were sacrificed on day 25 and tumor-infiltrating $CD4^+$ T cells were harvested and tested for expression of IL-17A and RORγt. Each dot represents data from a single mouse. Experiments were repeated 3 times.

(K) CD11c.DTR bone marrow chimeric mice were administered PDA tumor cells via a splenic injection. DT or PBS were serially administered beginning on day 12 after tumor establishment. Mice were sacrificed on day 25 and tumor-infiltrating $CD4^+$ T cells were harvested and tested for expression of IL-17A, IL-17F, and RORγt. This experiment was repeated twice (n=3-5/group; *$p<0.05$, **$p<0.01$, ***$p<0.001$, ****$p<0.0001$, *t*-test), SEM.

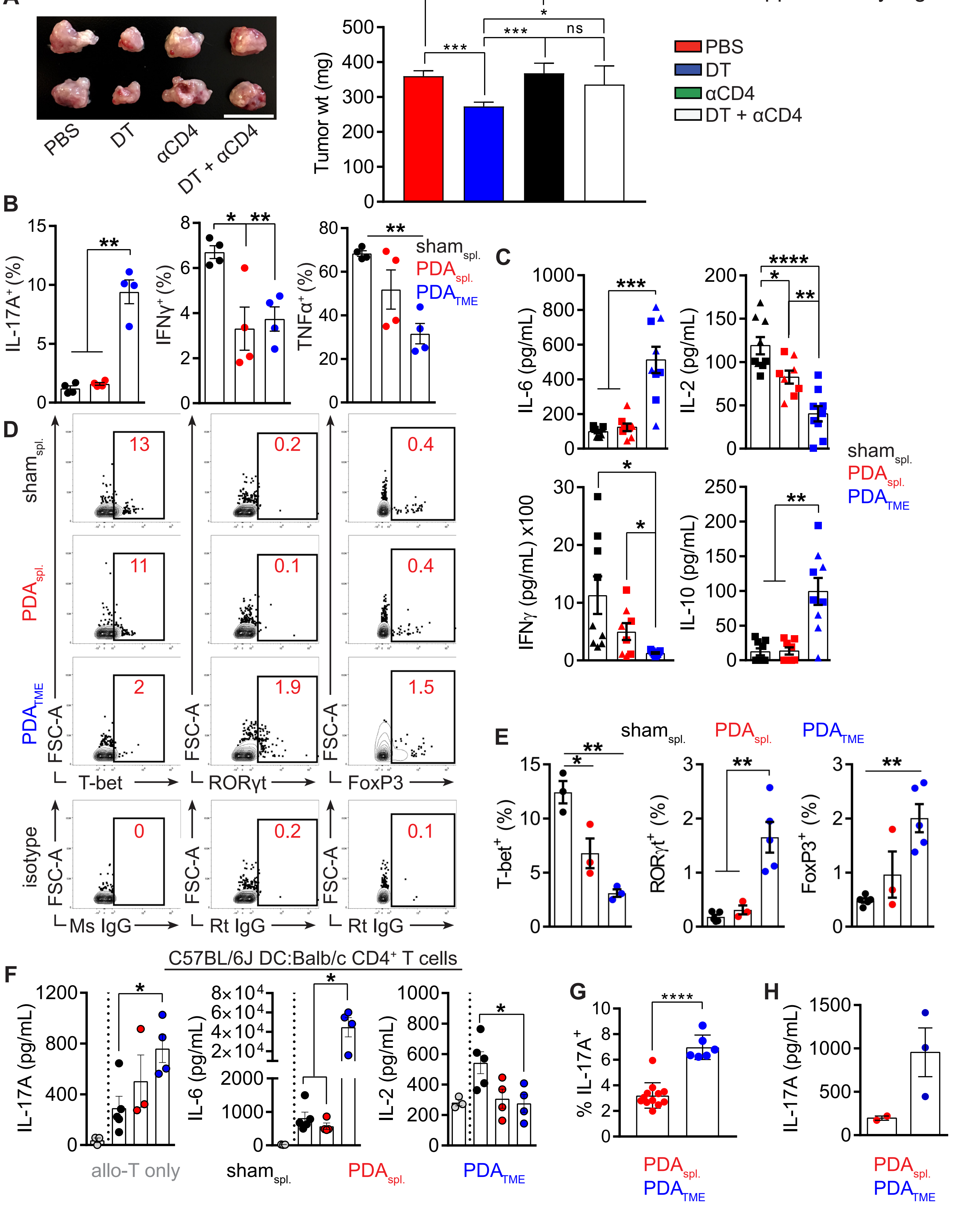
A
PBS
DT
αCD4
DT + αCD4
Tumor wt (mg)
ns
*
***
***
ns
PBS
DT
αCD4
DT + αCD4
B
IL-17A+ (%)
IFNγ+ (%)
TNFα+ (%)
sham spl.
PDA spl.
PDA TME
C
IL-6 (pg/mL)
IL-2 (pg/mL)
IFNγ (pg/mL) x100
IL-10 (pg/mL)
D
sham spl.
PDA spl.
PDA TME
isotype
FSC-A
T-bet
RORγt
FoxP3
Ms IgG
Rt IgG
13
0.2
0.4
11
0.1
0.4
2
1.9
1.5
0
0.2
0.1
E
T-bet+ (%)
RORγt+ (%)
FoxP3+ (%)
F
C57BL/6J DC:Balb/c CD4+ T cells
IL-17A (pg/mL)
IL-6 (pg/mL)
IL-2 (pg/mL)
allo-T only
G
% IL-17A+
H
IL-17A (pg/mL)

Supplemental Figure 2. PDA_{TME} DC promote tumorigenesis in a $CD4^+$ T cell dependent manner and elicit distinct $CD4^+$ T cell differentiation.

(A) CD11c.DTR bone marrow chimeric mice were challenged with orthotopic PDA and serially treated with DT or PBS beginning on day 12. Cohorts were additionally treated with a neutralizing mAb targeting $CD4^+$ T cells or isotype control also starting on day 12. Mice were sacrificed on day 25. Representative gross images of tumors and quantitative analysis of tumor weights are shown. This experiment was performed twice with similar results (n=5-10/group; scale bars = 1μm).

(B-E) $Ova_{323-339}$ peptide-pulsed PDA_{TME}, $PDA_{spl.}$, and $sham_{spl}$ DC were co-cultured with Ova-restricted $CD4^+$ T cells at a 1:5 ratio for 5 days. (B) $CD4^+$ T cell expression of IL-17A, IFNγ, and TNFα were determined using intracellular cytokine analysis on flow cytometry. (C) IL-6, IL-2, IFNγ, and IL-10 cytokine concentrations were measured in co-culture supernatants. Squares and triangles denote data from individual mice, pooled from two independent experiments. (D-E) Flow cytometric analysis of T_H-cell transcriptional regulators in Ova-restricted T_H-cells after co-culture with $Ova_{323-339}$ peptide-pulsed PDA_{TME}, $PDA_{spl.}$, or $sham_{spl}$ DC. Representative (D) and quantitative (E) data are shown. Results are representative of 5 independent experiments.

(F) PDA_{TME}, $PDA_{spl.}$, and $sham_{spl}$ DC from C57BL/6 mice were co-cultured with allogeneic $CD4^+$ T cells from Balb/c mice at a 1:5 ratio for 5 days in a mixed lymphocyte reaction. Cytokine concentrations were measured in cell culture supernatants. This experiment was performed twice.

(G) DCs harvested from tumor and spleen of 3-month-old KC mice were co-cultured with OT-II $CD4^+$ T cells at a 1:5 ratio for 5 days. T cells were gated on flow cytometry and tested for expression of IL-17A and IFNγ. This experiment was performed twice.

(H) DCs harvested from tumor and spleen of 3-month-old KPC mice were co-cultured with OT-II $CD4^+$ T cells at a 1:5 ratio for 5 days. IL-17A and IFNγ concentrations were measured in cell culture supernatant. This experiment was repeated twice (*$p<0.05$, **$p<0.01$, ***$p<0.001$, ****$p<0.0001$, *t*-test), SEM.

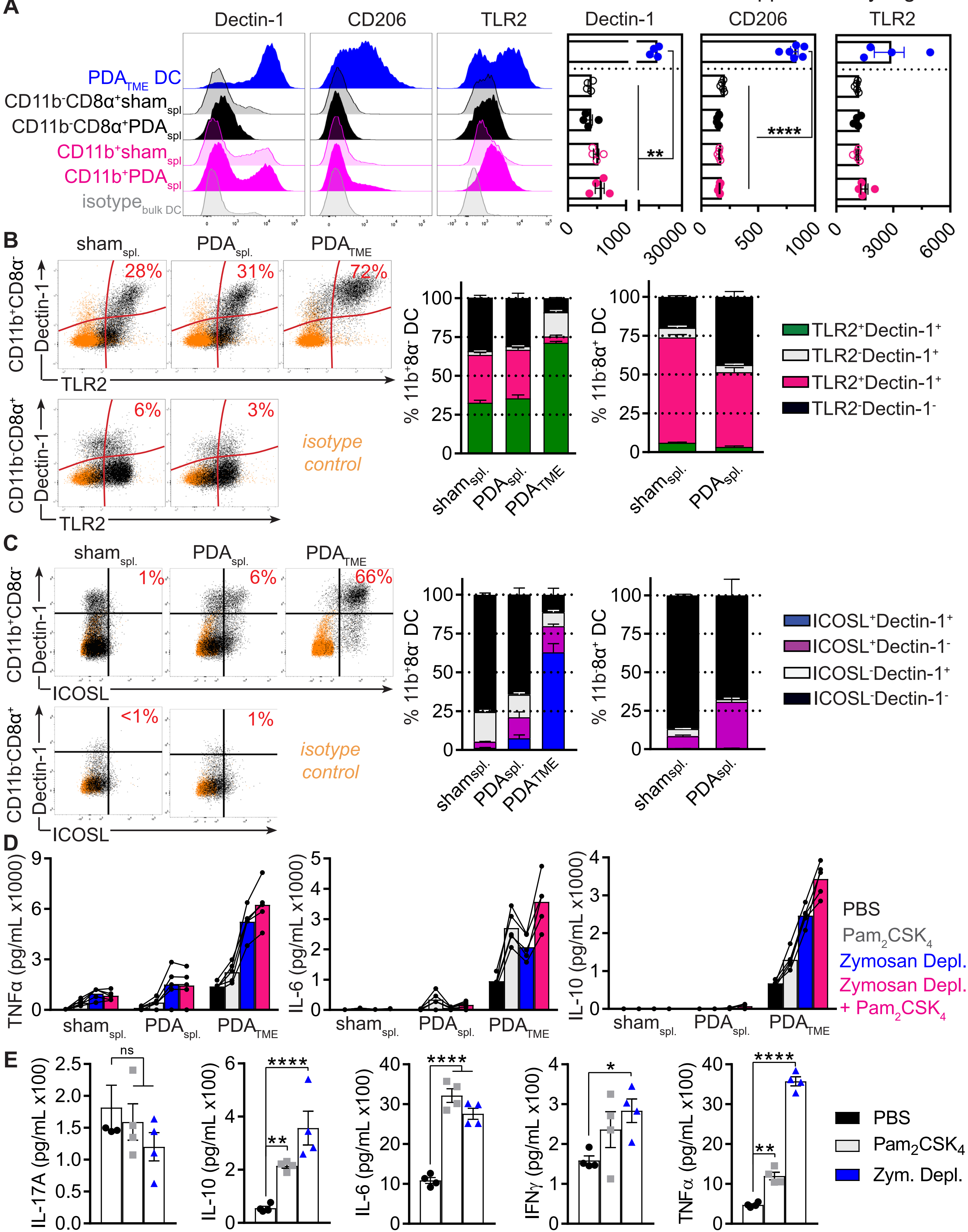
A
Dectin-1
CD206
TLR2
PDA TME DC
CD11b-CD8α+sham spl
CD11b-CD8α+PDA spl
CD11b+sham spl
CD11b+PDA spl
isotype bulk DC
**
****
B
sham spl.
PDA spl.
PDA TME
28%
31%
72%
6%
3%
CD11b+CD8α- Decitn-1
CD11b-CD8α+ Decitn-1
TLR2
isotype control
% 11b+8α- DC
% 11b-8α+ DC
TLR2+Dectin-1+
TLR2-Dectin-1+
TLR2+Dectin-1+
TLR2-Dectin-1-
C
1%
6%
66%
<1%
1%
ICOSL
ICOSL+Dectin-1+
ICOSL+Dectin-1-
ICOSL-Dectin-1+
ICOSL-Dectin-1-
D
TNFα (pg/mL x1000)
IL-6 (pg/mL x1000)
IL-10 (pg/mL x1000)
PBS
Pam2CSK4
Zymosan Depl.
Zymosan Depl. + Pam2CSK4
E
IL-17A (pg/mL x100)
IL-10 (pg/mL x100)
IL-6 (pg/mL x100)
IFNγ (pg/mL x100)
TNFα (pg/mL x100)
ns
**
****
*
PBS
Pam2CSK4
Zym. Depl.

Supplemental Figure 3. DC in PDA exhibit unique responses to PRR ligation.

(A) PDA_{TME} DC and $CD11b^{+}CD8\alpha^{-}$ and $CD8\alpha^{+}CD11b^{-}$ $PDA_{spl.}$ and $sham_{spl}$ DC were tested for expression of Dectin-1, CD206, and TLR2. Representative histograms and quantitative data based on MFI are shown. Gating strategy for each marker was determined by the fluorescence of the respective isotype control (not shown). Experiments were repeated more than 3 times.

(B) Comparison of co-expression of Dectin-1 and TLR2 in PDA_{TME} DC and $CD11b^{+}CD8\alpha^{-}$ and $CD8\alpha^{+}CD11b^{-}$ $PDA_{spl.}$ DC and $sham_{spl.}$ DC. Representative dot plots and quantitative data are shown. Orange dots indicate staining using isotype control. Data represents one of >3 independent experiments with n=4-7, showing similar results.

(C) Comparison of co-expression of Dectin-1 and ICOSL in PDA_{TME} DC and $CD11b^{+}CD8\alpha^{-}$ and $CD8\alpha^{+}CD11b^{-}$ $PDA_{spl.}$ DC and $sham_{spl.}$ DC. Representative and quantitative data are shown. Orange dots indicate staining using isotype control. Data represents one of >3 independent experiments (n=4-7).

(D) Bulk PDA_{TME}, $PDA_{spl.}$ DC, and $sham_{spl.}$ DC were cultured at a density of $5x10^{5}$ cells/ml with either PBS, Dectin-1 ligand (Zymosan depleted), TLR2 ligand (Pam_2CSK_4), alone or in combination. Cytokine concentrations in cell culture supernatant were determined at 36h. Experiments were repeated 3 times.

(E) PDA_{TME} DC were cultured with PBS, Zymosan depleted, or Pam_2CSK_4, loaded with $Ova_{323-339}$ peptide, and used to stimulate OT-II T cells (1:5 ratio). T cell expression of IL-17A, IL-10, IL-6, IFNγ, and TNFα was assessed at 5 days. Data represent one of 3 independent experiments with n=3-5, showing similar results (*p<0.05, **p<0.01, ****p<0.0001, *t*-test), SEM.

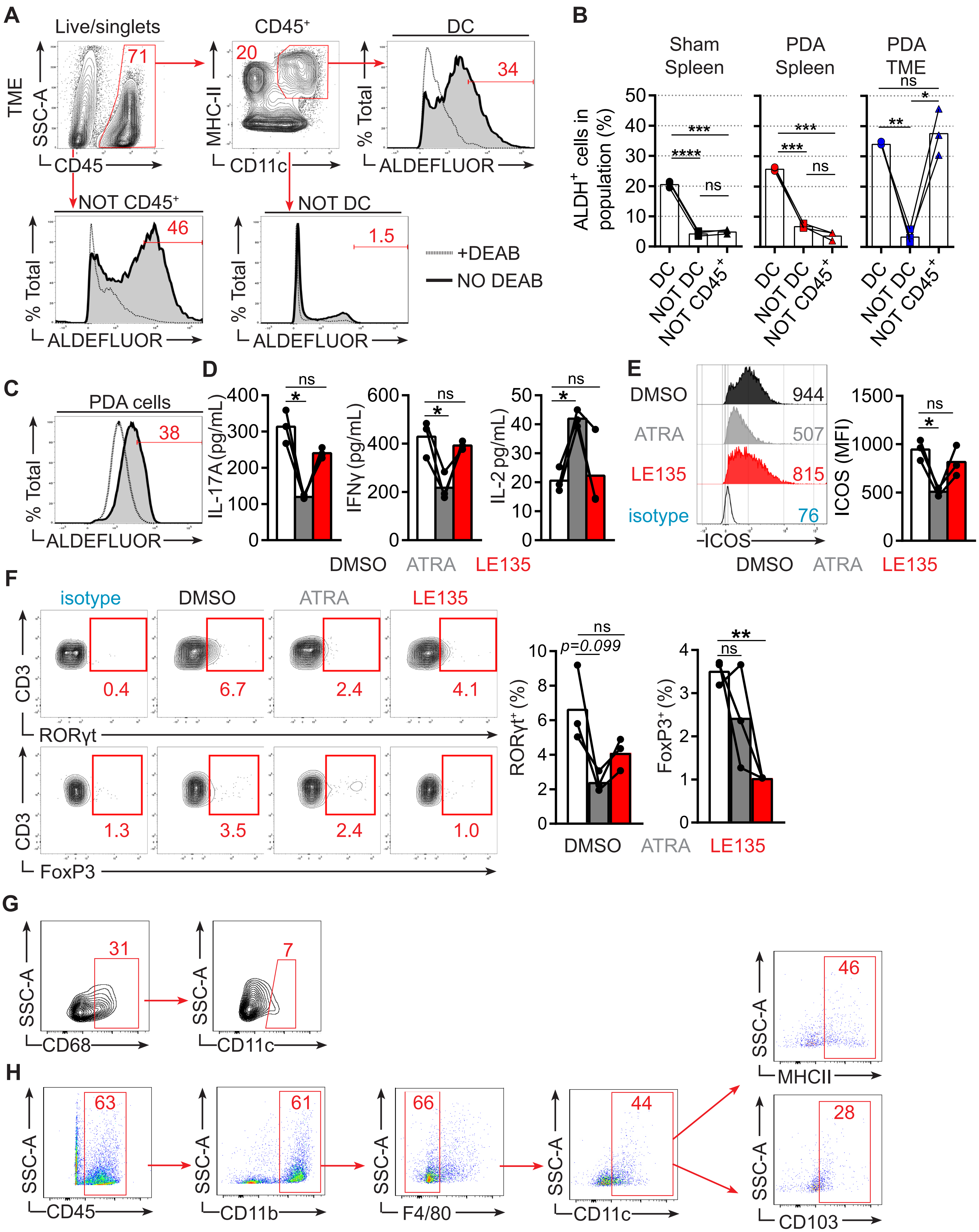
A
Live/singlets
TME
SSC-A
CD45
71
CD45+
MHC-II
CD11c
20
DC
% Total
ALDEFLUOR
34
NOT CD45+
46
NOT DC
1.5
+DEAB
NO DEAB
B
Sham Spleen
PDA Spleen
PDA TME
ALDH+ cells in population (%)
DC
NOT DC
NOT CD45+
ns
C
PDA cells
38
D
IL-17A (pg/mL)
IFNγ (pg/mL)
IL-2 pg/mL)
DMSO
ATRA
LE135
E
isotype
944
507
815
76
ICOS
ICOS (MFI)
F
RORγt
CD3
FoxP3
0.4
6.7
2.4
4.1
1.3
3.5
2.4
1.0
p=0.099
RORγt+ (%)
FoxP3+ (%)
G
CD68
31
CD11c
7
H
CD45
63
CD11b
61
F4/80
66
44
MHCII
46
CD103
28

Supplemental Figure 4. Retinoic Acid modulates PDA_{TME} DC-mediated T_H differentiation.

(A, B) Enzymatic activity of ALDH in PDA_{TME} DC, non-inflammatory cells ("NOT $CD45^+$"), and non-DC leukocytes ("NOT DC") was determined by the ALDEFLUOR assay. Representative (A) and quantitative (B) data including $Sham_{Spl.}$ and $PDA_{Spl.}$ controls are shown. Gates for each biological sample (shaded, solid histogram) were determined by the fluorescence of their respective DEAB controls (dotted histogram) and reflect the $ALDH^+$ frequencies quantified. This experiment was repeated twice.

(C) Enzymatic activity of ALDH in cultured KPC-derived PDA tumor cells was determined by the ALDEFLUOR assay. This experiment was repeated twice.

(D) Concentrations of supernatant cytokines in $CD4^+$ OT-II T cells co-cultures with Ova-pulsed PDA_{TME} DCs for 96h in the presence of either a vehicle (DMSO), ATRA, or LE135. This experiment was repeated twice.

(E) Representative and quantitative flow cytometric data on ICOS expression in $CD4^+$ T cells from PDA_{TME} DC co-cultures in (D) are shown. This experiment was repeated twice.

(F) Representative and quantitative flow cytometric data on RORγt and FoxP3 expression in $CD4^+$ T cells from PDA_{TME} DC co-cultures in (D). Representative and quantitative data are shown. This experiment was repeated twice.

(G) Representative flow cytometric data showing expression of CD11c on tumor associated macrophages in orthotopic PDA tumors. This experiment was repeated more than 5 times.

(H) $CD45^+$ leukocytes infiltrating orthotopic PDA tumors were tested for CD11b, F480, CD11c, MHC II, and CD103 expression. This experiment was repeated more than 5 times (*p<0.05, **p<0.01, ***p<0.001, ****p<0.0001, *t*-test), SEM.

Supplementary Figure 5

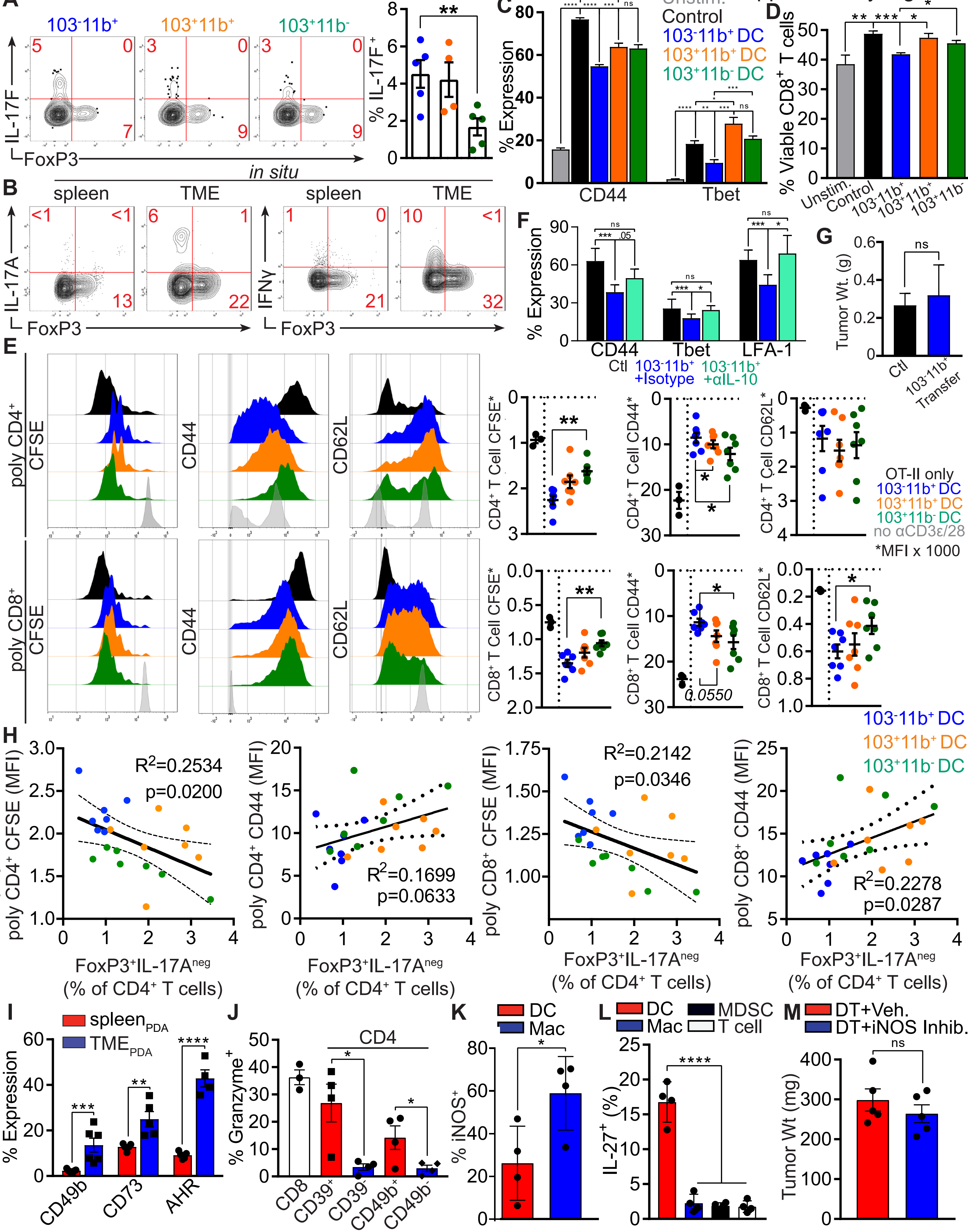

Supplemental Figure 5. $CD4^+$ T cells differentiated by select subsets of PDA-infiltrating DC are immune-suppressive.

(A) Ova-restricted $CD4^+$ T cells that were selectively entrained for 8 days by $Ova_{323-339}$ peptide-pulsed PDA_{TME} DC subsets were tested for co-expression of IL-17F and FoxP3. Representative contour plots and quantitative data are shown. This experiment was reproduced more than 3 times.

(B) $CD4^+$ T cells from tumor or spleen of PDA-bearing mice were assessed for co-expression of IL-17A / FoxP3 and IFNγ / FoxP3. Representative data are shown. This experiment was reproduced more than 5 times.

(C) Naive polyclonal $CD8^+$ T cells isolated from the spleens of WT mice were cultured for 96h on αCD3ε/αCD28 coated plates alone or in the presence of OT-II $CD4^+$ T cells harvested from OT-II $CD4^+$ T cell / PDA_{TME} DC subset co-cultures. Additional control polyclonal $CD8^+$ T cells were unstimulated. $CD8^+$ T cells were assessed by flow cytometry for expression of CD44 and Tbet. This experiment was repeated 4 times.

(D) Naive polyclonal $CD8^+$ T cells isolated from the spleens of WT mice were cultured for 96h on αCD3ε/αCD28 coated plates alone or in the presence of OT-II $CD4^+$ T cells harvested from OT-II $CD4^+$ T cell / PDA_{TME} DC subset co-cultures. Additional control polyclonal $CD8^+$ T cells were unstimulated. $CD8^+$ T cells were assessed by flow cytometry using cell death markers. This experiment was repeated twice.

(E) CFSE-labeled naive $CD8^+$ or $CD4^+$ polyclonal T cells isolated from the spleens of WT mice were cultured for 96h on αCD3ε/αCD28 coated plates in the presence of conditioned media from either OT-II $CD4^+$ T cells cultured alone or select OT-II $CD4^+$ T cell / PDA_{TME} DC subset co-cultures. Polyclonal $CD8^+$ and $CD4^+$ T cells were assessed by flow cytometry for CFSE-dilution,

CD62L expression, and CD44 expression. Representative and quantitative plots are shown. Each individual well from the OT-II / PDA_{TME} DC subset co-cultures was used to generate conditioned media for a single well of polyclonal $CD8^+$ and $CD4^+$ T cells.

(**F**) Naive splenic WT polyclonal $CD8^+$ T cells were cultured for 96h on αCD3ε/αCD28 coated plates in the presence of vehicle or in co-culture with OT-II $CD4^+$ T cells entrained by $CD103^-$ $CD11b^+$ PDA_{TME} DC, either alone or with a neutralizing αIL-10 mAb. $CD8^+$ T cells were assessed by flow cytometry for expression of CD44, Tbet, and LFA-1. This experiment was repeated 3 times (n=5/group).

(**G**) KPC-derived tumor cells were orthotopically implanted in pancreata of IL-$10^{-/-}$ mice admixed with either PBS or $CD103^-CD11b^+$ DC_{TME} and sacrificed at 3 weeks. Quantitative data on tumor weight are shown (n=5/group).

(**H**) Scatter plots correlating the phenotypes of polyclonal T cells receiving the conditioned media described in (E) to the phenotype of the T_H-cells of the respective OT-II / PDA_{TME} DC subset co-culture generating the conditioned media. Linear regression was used to determine the best-fit line (solid) and displayed with 95% confidence intervals (dotted-lines); p-values indicate significance of a non-zero slope, determined by an F-test.

(I) Tumor and spleen were harvested on day 25 from mice bearing orthotopic PDA. Tumor-infiltrating and splenic $CD4^+$ T cells were assessed for expression of CD49b, CD73, and AHR. Representative and quantitative data are shown. This experiment was repeated more than 5 times.

(J) Orthotopic PDA tumor was harvested from WT mice on day 25. Tumor-infiltrating $CD8^+$ T cells and subsets of $CD4^+$ T cells were assessed for expression of Granzyme B. This experiment was repeated twice.

(K) Orthotopic PDA tumor was harvested from WT mice on day 25. DC_{TME} and tumor-associated macrophages were tested for expression of iNOS by flow cytometry. This experiment was repeated twice.

(L) Orthotopic PDA tumor was harvested from WT mice on day 25. DC_{TME}, F4/80$^+$ tumor-associated macrophages, Gr1$^+$CD11b$^+$ myeloid-derived suppressor cells (MDSC), and T cells were tested for expression of IL-27 by flow cytometry. This experiment was repeated twice.

(M) WT mice were made chimeric using bone marrow from CD11c.DTR mice. Cohorts were challenged with orthotopic PDA 7 weeks later. Mice began serial treatment with DT as well as an iNOS inhibitor or vehicle on day 12. Tumor weight was measured at 25 days. This experiment was repeated twice (n=5; *p<0.05, **p<0.01, ***p<0.001, ****p<0.0001, *t*-test), SEM.

Supplementary Figure 6

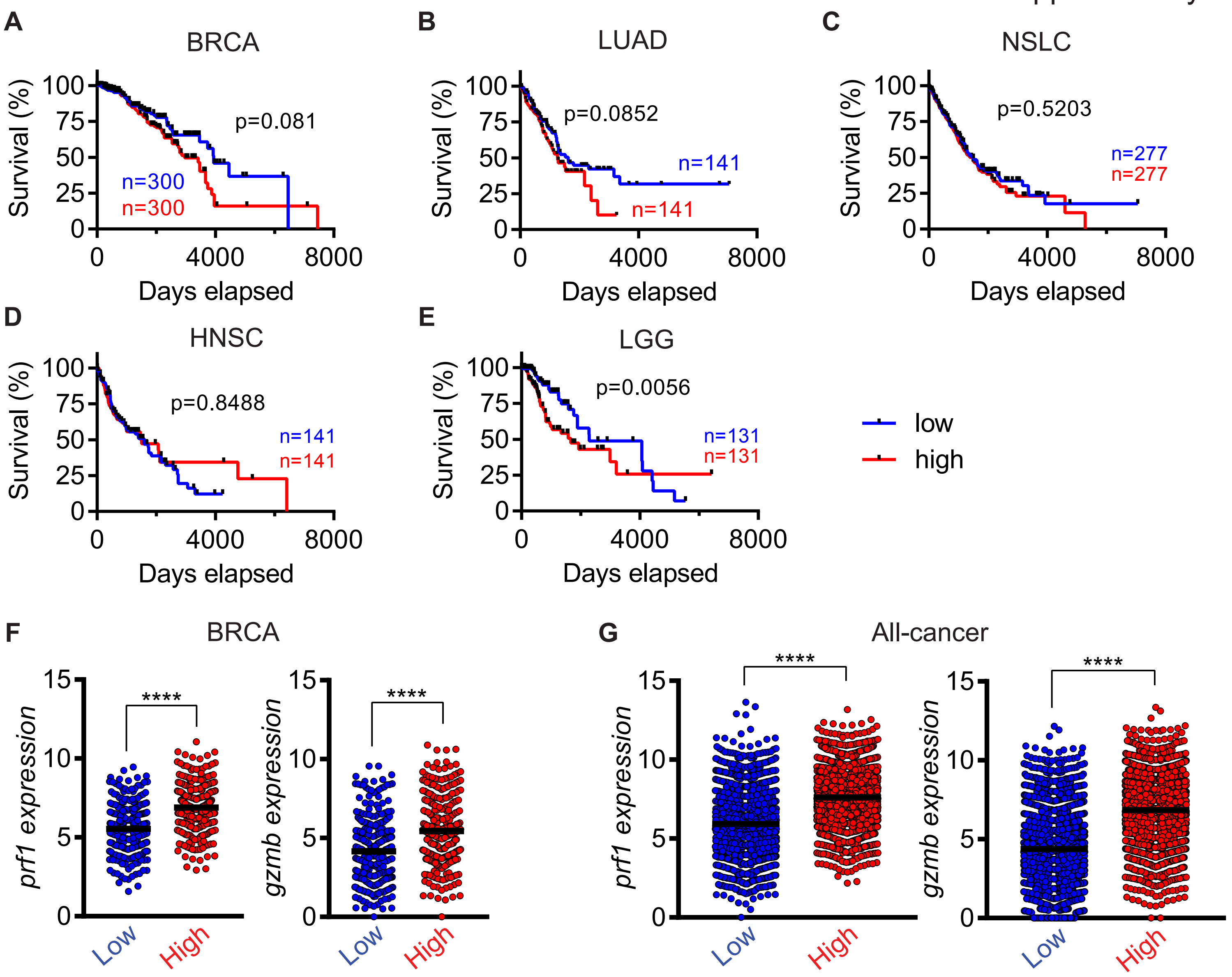

Supplemental Figure 6. CD4^{+} T programming is distinct in specific human cancers.

(A-E) Patients from the TCGA database were stratified on the basis of validation signature scores (described in Figure 6) derived from tumor RNAseq gene expression (PolyA+ IlluminaHiSeq). Kaplan-Meier survival data comparing the highest (red) and lowest (blue) quartiles of patients from the BRCA (A), LUAD (B), NSLC (C), HNSC (D), and LGG (E) datasets are shown. P-values were calculated using the log-rank (Mantel-Cox) method.

(F, G) Plots represent *prf1* and *gzmb* RNAseq gene expression in the tumors of BRCA patients (F) and all cancers combined (G) from the highest (red) and lowest (blue) quartiles of the ΣTr1 - ΣTREG validation signature using RNAseq data from the TCGA database. All dot plots were analyzed with unpaired t tests with Welch's correction (****$p<0.0001$).
